# Navigating phenylketonuria management to improve it in Latin America: a systematic literature review and applicability analysis

**DOI:** 10.3389/fnut.2024.1390133

**Published:** 2024-06-25

**Authors:** Alex S. Aguirre, Edison Haro, Alberto Campodónico, Alissa Mendoza, Bernarda Bahamonde, Vanessa I. Romero

**Affiliations:** School of Medicine, Universidad San Francisco de Quito, Quito, Ecuador

**Keywords:** phenylketonuria, Latin America, nutrition, guidelines, multidisciplinary care, challenges

## Abstract

**Introduction:**

Phenylketonuria (PKU) is an autosomal recessive metabolic disorder resulting from phenylalanine hydroxylase deficiency, which impacts neurodevelopment. Lifelong low-phenylalanine diets and multidisciplinary care are pivotal for managing PKU. Latin American challenges in PKU care include diverse newborn screening programs, limited specialized healthcare, and resource scarcity.

**Methods:**

A systematic literature review was conducted (2010–2023) on PKU management following PRISMA guidelines. Inclusion criteria encompassed English/Spanish articles focusing on PKU management guidelines approved by an organization as well as articles focusing on PKU management in Latin America. After screening 127,276 results, 6 articles were included.

**Results:**

Six articles were analyzed, highlighting shared principles like multidisciplinary care, lifelong dietary adherence, personalized plans, and regular monitoring. Guides emphasized regional variations, breastfeeding complexities, and challenges for pregnant women with PKU.

**Discussion:**

Multidisciplinary care emerges as critical, incorporating physicians, psychologists, dietitians, nurses, and genetic counselors. Lifelong adherence to low-phenylalanine diets and personalized strategies for different life stages are emphasized. Challenges in Latin America include healthcare gaps, scarce resources, and reliance on international guidance. The importance of breastfeeding, preconception care, and comprehensive support for pregnant women with PKU is underscored.

**Conclusion:**

Collaborative efforts are essential to address PKU challenges in Latin America. Advocacy for awareness, specialized training, regional databases, and international collaborations can enhance diagnosis and management, ensuring a better quality of life for PKU individuals in the region. Embracing lessons from existing guides will contribute to improved PKU care and overall well-being.

## 1 Introduction

Phenylketonuria (PKU) is an autosomal recessive innate error of metabolism due to a deficiency in the enzyme phenylalanine hydroxylase, produced in the liver (1). This enzyme is crucial for converting phenylalanine (phe) to tyrosine (tyr), a vital component in both fetal and adult neurodevelopment (1, 2). The primary treatment for PKU involves a lifelong low-phe diet with phe supplementation to prevent neurotoxicity, as elevated phe levels can lead to irreversible intellectual disability and various complications, including seizures, behavioral disorders, eczema, and gait instability (1–3). Nutritional assessment for PKU patients includes restrictions on animal-based foods and limitations on other items such as sweet potatoes (4). Supplementation with tyr, essential amino acids, vitamins, and minerals is essential, requiring biweekly blood phe and tyr quantification for optimal management, along with strict diet assessment and clinical evaluation by specialists (1, 2).

PKU is commonly included in newborn screening programs. However, there is considerable diversity in the implementation of these programs across Latin America (5). This lack of comprehensive screening is associated with higher infant mortality rates in the region, currently standing at 14.0 per 1,000 live births (6, 7). Challenges include the absence of national newborn screening registries, hampering case contact, and geographic and economic burdens limiting program accessibility. Latin American countries face difficulties keeping pace with the rapid evolution of newborn screening techniques observed in developed nations (5). A shared objective should be the prompt detection, diagnosis, treatment, and follow-up of newborn screening-related diseases, with tailored treatment guidelines addressing economic, social, and geographic considerations (3, 6, 7). This is especially critical in countries like Ecuador, where public institutions lack specialization in the medical and nutritional treatment required for PKU patients, necessitating knowledge of nutrition by physicians and families. The diverse foods in Latin America, exemplified by the four regions in Ecuador such as the different types of fruits encountered in the coast region comparted to the ones in the highlands, make it challenging to establish national guidelines or databases for quantifying essential amino acids, such as phe and tyr, vital for proper PKU control. While Ecuador initiated a neonatal screening program for PKU in 2012, challenges persist in accessing healthcare systems and timely diagnosis, contributing to a high prevalence of intellectual disability in certain communities (8).

For optimum patient outcomes, PKU requires a multidisciplinary and personalized management. This literature review explores the multifaceted landscape of PKU care, particularly focusing on the Latin American context. The reviewed materials encompass a series of guides offering diverse perspectives on PKU management, emphasizing various aspects such as multidisciplinary team approaches, dietary interventions, regular monitoring, and regional food variations. In addition, demonstrates the challenges as limited resources, lack of specialized professionals and patient education. We aim to contribute meaningful insights to the advancement of PKU care in the Latin American healthcare landscape (1, 2, 6, 7).

## 2 Methods

### 2.1 Systematic literature review

**Figure 1:**
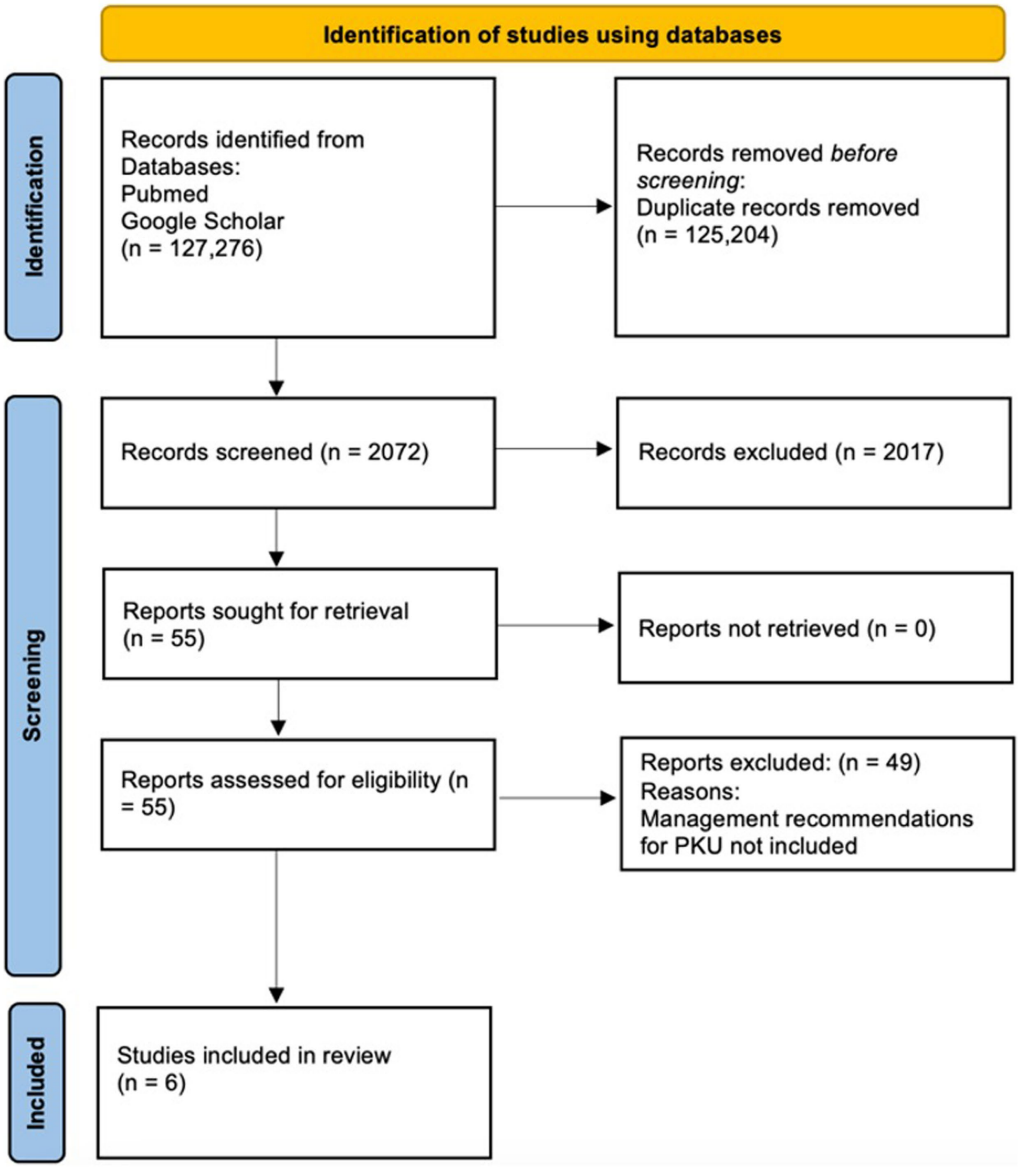
PRISMA protocol for this study. Identification, screening, and inclusion process.

The present study is a systematic literature review of the nutritional management of phenylketonuria in the world and Latin America. We carried out this systematic review using the PRISMA protocol. Figure 1 shows the results of the study using this protocol. Inclusion criteria were established for selecting the adequate studies. Electronic databases, such as PubMed and Google Scholar, were used to search articles and clinical guidelines addressing the nutritional management of phenylketonuria in Latin America and across the world. We performed a systemic literature review based on articles from 2010 to 2023 using the key words “Phenylketonuria,” “PKU,” “Latin America,” “Nutrition,” “Guidelines,” “Nutritional,” “Assessment,” and “Recommendations.”

### 2.2 Inclusion and exclusion criteria

Inclusion criteria were English and Spanish articles published in peer-reviewed journals from January 2010 to June 2023 as well as publications focusing on the nutritional management of phenylketonuria worldwide approved by an organization in the form of guidelines and also articles that showed the management of PKU in Latin America.

### 2.3 Selection and information extraction

A broad search of PubMed and Google Scholar yielded 127,276 results. After removing duplicate and irrelevant articles based on title/abstract screening, 55 full-text articles were assessed for eligibility and were screened against the inclusion criteria. Finally, 6 articles were included in this systematic literature review, all written in English as represented in Figure 1.

## 3 Results

Table 1 shows each article and offers unique perspectives, contributing valuable knowledge to guide the holistic understanding and management of PKU.

**Table 1:** Recommendations and applicability.

Authors	Summarized recommendations	Based on guidelines/practices
MacLeod et al. (1)	Management should be accomplished by a multidisciplinary team (healthcare professionals including a physician, psychologist, metabolic dietitian, nurse, and genetic counselor). Management should be individualized. The goal of the management should be to achieve the target level according to the patient’s age and requirements. Medical treatment can be used when there is poor compliance to diet, such as BH4, LNAAs, and glycomacropeptide.	Europe and US
Vockley et al. (2)	NBS for PAH deficiency in the United States is now primarily performed by tandem mass spectrometry. Quantitative blood aminoacids should be performed as part of the diagnostic testing for follow-up of a positive NBS. Additional testing is needed such as analysis of pterin metabolism. PAH genotyping is indicated for improved therapy planning. Sapropterin may be useful in reducing PHE levels in responsive patients. Treatment for PAH deficiency should be lifelong for patients with untreated PHE levels >360 umol/L. Maintaining a treated PHE level of 120–360 umol/L is recommended for all patients of all ages. Fetal development is optimal when maternal PHE levels are <360 umol/L prior to conception. Mothers with PAH deficiency may safely breastfeed. Mothers with PAH deficiency should maintain a Phe-restricted diet. Genetic counseling should be provided for individuals with PAH deficiency and their families. Appropriate intellectual and mental health assessments are important for individuals affected with PKU.	US
van Wegberg et al. (9) and van Spronsen et al. (10)	NBS is a national obligation but requires: a robust infrastructure in which blood can be taken from all newborns (ideally between 24 and 72 h after birth) and a laboratory that can handle bloodspots efficiently. Low-income countries may consider using the NBS laboratory facilities of other countries. BH4 deficiencies must be excluded. Patient genotyping is important. All patients with phe > 360 umol/L should be treated. Treatment should start as soon as possible during the first 10 days of age. In treated PKU patients up to the age of 12 years, target Phe levels should be 120–360 umol/L. In treated PKU patients aged >12 years the target Phe levels should be 120–600 umol/L. Phe measurement should be taken weekly until the first year, then fortnightly until 12 years, and monthly after 12 years. If planning on pregnancy: pre-pregnancy weekly and during pregnancy twice weekly. Women with untreated blood Phe values <360 umol/L do not require treatment to lower blood Phe before or during pregnancy. In treated pregnant PKU patients the target Phe levels should be 120–360 umol/L. The time between blood Phe sampling and the patient/parent receiving the result should be less than 5 days. Neurocognitive evaluations should be performed at 12 and 18 years of age in all patients. In patients younger than 12 years, when more than 50% of the Phe concentrations are out of target range over a period of 6 months, consider: Increased frequency of blood phe monitoring and outpatient visits with re-education. Psychology consultation or social worker intervention. Hospital admission. In patients younger than 12 years, when around 100% of blood Phe concentrations are out of target range over a period of 6 months and there are other signs of failure of adherence, such as lack of cooperation, clinic non-attendance, or unresolved issues outside PKU consider: consultation with social services and child safeguarding measures.	Europe
MacDonald et al. (11)	Phe intake should be divided throughout the day. Encourage the use of fruit and vegetables containing phe ≤75 mg/100 g. Try and give 5 portions/day with at least one portion at each meal. A portion of fruits and vegetables is defined as a handful (using the patient’s hand size as a guide). Encourage special low protein foods such as bread and pasta at most meals to provide calories, aid satiety, and variety. Breast milk mas advantages compared to standard infant formula. Promote the use of plant milks instead of animal milk, vegetable foods/plant foods to replace and higher protein foods, and the use of spices, seasonings and sauces to help improve the palatability and acceptance of vegetable dishes. Meet with the PKU team 4 to 6 months prior to their anticipated start of pregnancy to achieve and maintain phe levels prior to conception. If women do become pregnant unexpectedly or suspect they are pregnant, they should be given an emergency card with a guide on the actions they should take. Patients should be given an emergency box consisting of a 7-day supply of protein substitute, essential low protein special foods, blood phe monitoring equipment, low phe diet information, low protein recipes and important contact details for the PKU team.	Europe
Poloni et al. (6)	There is no agreement on when to start dietary therapy. However, most than half of the countries use the ≥360 umol/L cutoff. There is no agreement on Phe target levels. However, most countries use 120–140 umol/L until 2 years of age and pregnancy, then 120–360 umol/L over 2 years of age. Almost all countries except for Dominican Republic have a NBS program analyzing dried blood spots via fluorometric assay. Nutritional management is challenging because only 9 out of the 13 countries have guidelines, but most of them use international guidelines due to lack of national management protocols and regional database of Phe content of foods. Sapropterin is only available in Argentina, Brazil, Costa Rica, Dominican Republic and Mexico. LNAAs are only available in Argentina and Peru. Glycomacropeptide is only available in Argentina.	Brazil, Argentina, Colombia, Venezuela, Costa Rica, Chile, Mexico, Paraguay, Peru, Dominican Republic, Panama, Uruguay, and Cuba.

Tetrahydrobiopterin (BH4), large neutral aminoacids (LNAAs), Newborn Screening (NBS), Phe (phenylalanine), PAH (phenylalanine hydroxylase).

### 3.1 Shared key points by all guidelines

All guidelines highlight the necessity of a multidisciplinary team approach, involving healthcare professionals like physicians, psychologists, dietitians, nurses, and genetic counselors depending on individual needs (1, 2, 6, 9–11). Another shared aspect is the importance of lifelong adherence to a low-Phe diet to prevent sequels. Personalized management strategies considering factors like age, growth rate, and blood Phe concentrations, are required for patients at different life stages (1, 2, 6, 9–11). Regular monitoring of blood Phe concentrations emphasizes the importance of tracking metabolic levels to adjust dietary plans effectively (3). Patient and family education empowers them to take control and make informed decisions. These shared principles underscore the necessity of a comprehensive and individualized approach to PKU management, reinforcing the importance of collaboration, continuity, and patient-focused care across the spectrum of metabolic disorders.

### 3.2 Unique key points from the practices in Latin America

Among the 13 countries examined by Poloni et al., only 9 indicated the presence of national guidelines for managing Phenylketonuria (PKU), while 12 out of 22 centers had local management protocols (6). However, a majority of respondents turned to international guidelines for theoretical guidance, possibly due to outdated or incomplete local guidelines. Monitoring practices varied widely, with the highest agreement (65%) for measuring phe once a week or more in infants younger than 1 year, aligning with both American and European recommendations. However, the decision on phe monitoring lacked uniformity and could have significant consequences. Only 18% of centers reported possessing an adequate regional database of Phe content in foods, while 33% had access to only an incomplete database. The remaining 49% of centers relied on various international nutritional resources to support patients, families, and professionals. While specially designed low-protein products proved effective in enhancing metabolic control, they posed a considerable financial burden to families (6).

## 4 Discussion

Managing PKU poses unique challenges requiring a comprehensive, individualized approach, with a central emphasis on a multidisciplinary healthcare team (1, 2, 9, 10). This collaborative model ensures holistic care, addressing nutritional, psychological, social, and genetic aspects. Long-term adherence to a low-phe diet is pivotal for positive outcomes and normal growth (1). Concerns about breastfeeding practices in PKU patients highlight the need for standardized guidelines, specialized training, and support for healthcare providers and families (9, 10, 12, 13). Meticulous monitoring and dietary adherence before conception are crucial due to elevated complication risks. Preconception dietary training is essential, promoting improved metabolic control for favorable maternal and infant outcomes (9). A multidisciplinary care approach involving geneticists, obstetricians, and pediatricians is vital, emphasizing teamwork in optimizing maternal and fetal health (1, 2, 9, 10).

The challenges faced by healthcare providers in Latin America in managing PKU patients are various. Limited access to specialized healthcare professionals and resources is a barrier, aggravated by a scarcity of awareness and familiarity with PKU among healthcare providers (6). This issue is worsened by inadequate opportunities for specialized training and continuing education, hindering the effective management of PKU patients. Laboratories capable of quantifying phe are primarily situated in major cities, resulting in inconsistent and expensive testing. The dietary management of PKU across Latin America varies, with a significant scarcity of experienced nutritional and medical professionals. A mere 45% of physicians and dietitians involved in PKU treatment have completed specialization courses, and only 9 out of 13 countries report having national guidelines for PKU management (6). The absence of region-specific databases detailing the phe content of foods further complicates dietary recommendations, potentially leading to suboptimal outcomes; thus, we are supplementing a list with available food products and their quantification (Supplementary Table S1) (6). In Ecuador specifically, the responsibility for PKU management falls on gastroenterologists and geneticists due to the absence of a dedicated multidisciplinary team. The scarcity of metabolic dietitians forces patients, and often parents, to seek guidance from other countries like Argentina and Chile (6). Access to commercial low-phe foods in Latin America, including Ecuador, is notably limited, with the exclusive option being formula, which is only available at the public system, and there is not a private option to buy it. Furthermore, there is a lack of dietitians with experience, and a deficit in dietary education for these patients. In the public system of Ecuador, patients are monitored for phe levels twice a year, receiving results 3 months post-sample collection. This situation is exacerbated by the lack of regional databases containing phe content information for foods and the absence of specially designed low-protein products and commercially available alternatives. Furthermore, there is not many options for pediatric patients with PKU in most supermarkets, this may worsen the problem in adolescent ages where they want to experiment with their environment. Moreover, there is a critical gap in comprehensive care for pregnant women with PKU in Ecuador, as there are no dedicated dietitians or a specialized healthcare team catering to this demographic. Addressing these challenges requires collaborative efforts from healthcare institutions, policymakers, and educators to enhance awareness, provide specialized training, establish robust regional databases, and facilitate international collaborations for knowledge exchange and resource availability (14–17).

PKU is included in the public free nationwide newborn screening program for all infants in Ecuador. A study using data from this screening program between 2012 and 2019 reported an incidence of PKU of approximately 1.53 per 100,000 live births (8). However, the study did not provide information on false-negative cases, highlighted discrepancies in coverage due to inadequate case detection in certain provinces and does not mention a follow-up strategy for the positive cases. In our experience, despite the free and supposedly nationwide nature of the newborn screening program, some patients, particularly in rural areas, were not detected, and there is no plan to detect older patients (before 2012). Additionally, there is a shortage of nutritionists experienced in managing the PKU diet, even in major cities. The Ministry of Health is the sole provider of the formula but does not supply phenylalanine-free food, and patients cannot buy on stores.

Countries like Argentina, Brazil, Chile, Colombia, Costa Rica, Ecuador, Guatemala, Mexico, and Peru are improving their healthcare systems in response to the United Nations’ call to action. Our study aims to highlight the significance of these efforts, raise awareness, and advocate for effective solutions. We propose several specific measures: developing region-specific management strategies tailored to the diverse healthcare landscapes within Latin America, which account for variations in healthcare infrastructure, access to resources, and cultural considerations to ensure PKU management protocols are effectively adapted to local contexts. We emphasize the importance of establishing standardized methods for consulting PKU patients and distributing PKU-free products, suggesting the implementation of telemedicine or online consultation services to reach patients in remote areas, alongside centralized procurement, and distribution systems to ensure equitable access to essential products across the region. Furthermore, we underscore the necessity of developing guidelines or algorithms based on individual patient needs, such as age, weight, dietary requirements, and PKU severity, to help healthcare providers make informed decisions on the quantity and type of products to allocate, thereby optimizing patient outcomes. Additionally, we propose establishing a South American computer network dedicated to the detection and management of PKU to facilitate communication and data sharing among healthcare professionals, researchers, policymakers, and patient advocacy groups, thus enhancing the efficiency of diagnosis, treatment, and follow-up care. We also advocate for the development of comprehensive follow-up programs tailored to different age groups, from infancy to pregnancy, to ensure ongoing monitoring, nutritional counseling, psychosocial support, and medical interventions as needed. Finally, we highlight the importance of establishing multidisciplinary teams to manage complex PKU cases associated with other acute or chronic conditions, ensuring that patients receive comprehensive and coordinated care.

In conclusion, this study highlights the urgent need for collaborative efforts between healthcare institutions, policymakers, and international organizations to bridge the gaps in PKU management in Latin America. Managing PKU in Latin America involves multifaceted approaches. Establishing and reinforcing multidisciplinary teams, including physicians, psychologists, dietitians, nurses, and genetic counselors, is essential for comprehensive care. By enhancing and fostering a supportive environment, we can make the way for improved diagnosis, effective management, and a better quality of life for individuals living with PKU in the region. Embracing the lessons from these guides, we can work towards a future where every PKU patient receives the care and support they deserve, ultimately leading to better health and well-being.

## Author contributions

AA: Conceptualization, Data curation, Formal analysis, Investigation, Methodology, Resources, Writing – original draft, Writing – review & editing. EH: Data curation, Formal analysis, Investigation, Methodology, Resources, Writing – original draft, Writing – review & editing. AC: Data curation, Formal analysis, Investigation, Methodology, Resources, Writing – original draft, Writing – review & editing. AM: Data curation, Formal analysis, Investigation, Methodology, Resources, Writing – original draft, Writing – review & editing. BB: Conceptualization, Data curation, Formal analysis, Investigation, Methodology, Resources, Writing – original draft, Writing – review & editing. VR: Conceptualization, Data curation, Formal analysis, Investigation, Methodology, Project administration, Resources, Supervision, Writing – original draft, Writing – review & editing.
