# Supplementary material for: Navigating phenylketonuria management to improve it in Latin America: a systematic literature review and applicability analysis
Source: Front Nutr. 2024 Jun 25;11:1390133. doi: 10.3389/fnut.2024.1390133 (PMC11236298; doi:10.3389/fnut.2024.1390133)
Supplement: Supplementary file 1 [file Table_1.DOCX]

Supplementary Material

# Supplementary Table

| **Name Spanish** | **Name English** | **protein** | **total fat** | **carbs** | **energy** | **phe** | **tyr** |
| --- | --- | --- | --- | --- | --- | --- | --- |
|  |  | **g** | **g** | **g** | **kcal** | **g** | **g** |
| AMARANTO | AMARANTH,UNCKD | 13.56 | 7.02 | 65.25 | 371 | 0.542 | 0.329 |
| ARROZ PRECOCIDO | RICE,WHITE,LONG-GRAIN,PRECKD OR INST,ENR,DRY | 7.82 | 0.94 | 82.32 | 380 | 0.413 | 0.232 |
| ARROZ BLANCO GRANO CORTO | RICE,WHITE,SHORT-GRAIN,RAW,UNENR | 6.5 | 0.52 | 79.15 | 358 | 0.381 | 0.238 |
| ARROZ BLANCO GRANO CORTO (COCIDO) | RICE,WHITE,SHORT-GRAIN,CKD,UNENR | 2.36 | 0.19 | 28.73 | 130 | 0.144 | 0.09 |
| ARROZ BLANCO GRANO LARGO | RICE,WHITE,LONG-GRAIN,REG,RAW,UNENR | 7.13 | 0.66 | 79.95 | 365 | 0.398 | 0.216 |
| ARROZ BLANCO GRANO LARGO (COCIDO) | RICE,WHITE,LONG-GRAIN,REG,CKD,UNENR,WO/SALT | 2.69 | 0.28 | 28.17 | 130 | 0.153 | 0.086 |
| ARROZ BLANCO GRANO LARGO PARBOLIZADO | RICE,WHITE,LONG-GRAIN,PARBLD,UNENR,DRY | 7.51 | 1.03 | 80.89 | 374 | 0.353 | 0.221 |
| ARROZ BLANCO GRANO LARGO PARBOLIZADO (COCIDO) | RICE,WHITE,LONG-GRAIN,PARBLD,UNENR,CKD | 2.91 | 0.37 | 26.05 | 123 | 0.127 | 0.08 |
| ARROZ BLANCO GRANO MEDIO | RICE,WHITE,MEDIUM-GRAIN,RAW,UNENR | 6.61 | 0.58 | 79.34 | 360 | 0.348 | 0.217 |
| ARROZ BLANCO GRANO MEDIO (COCIDO) | RICE,WHITE,MEDIUM-GRAIN,CKD,UNENR | 2.38 | 0.21 | 28.59 | 130 | 0.126 | 0.079 |
| ARROZ INTEGRAL GRANO LARGO | RICE,BROWN,LONG-GRAIN,RAW | 7.94 | 2.92 | 77.24 | 370 | 0.41 | 0.298 |
| ARROZ INTEGRAL GRANO LARGO (COCIDO) | RICE,BROWN,LONG-GRAIN,CKD | 2.58 | 0.9 | 22.96 | 111 | 0.133 | 0.097 |
| ARROZ INTEGRAL GRANO MEDIO | RICE,BROWN,MEDIUM-GRAIN,RAW | 7.5 | 2.68 | 76.17 | 362 | 0.387 | 0.281 |
| ARROZ INTEGRAL GRANO MEDIO (COCIDO) | RICE,BROWN,MEDIUM-GRAIN,CKD | 2.32 | 0.83 | 23.51 | 112 | 0.119 | 0.087 |
| AVENA (HOJUELAS) | OATS | 16.89 | 6.9 | 66.27 | 389 | 0.616 | 0.333 |
| AVENA MOLIDA QUAKER | CEREALS,QUAKER,QUICK OATS,DRY | 13.7 | 6.87 | 68.18 | 371 | 0.895 | 0.573 |
| CANGUIL | POPCORN,AIR-POPPED | 12.94 | 4.54 | 77.9 | 387 | - | - |
| CANGUIL DULCE DE CARAMELO | POPCORN,CARAMEL-COATED,WO/PNUTS | 3.8 | 12.8 | 79.1 | 431 | 0.7 | 0.358 |
| CEBADA | BARLEY,HULLED | 12.48 | 2.3 | 73.48 | 354 | 0.556 | 0.284 |
| CEBADA PERLADA | BARLEY,PEARLED,RAW | 9.91 | 1.16 | 77.72 | 352 | 0.488 | 0.183 |
| CENTENO | RYE | 10.34 | 1.63 | 75.86 | 338 | 0.435 | 0.2 |
| CEREAL DESAYUNO CON MIEL | CEREALS RTE,KELLOGG,KELLOGG'S HONEY CRUNCH CORN FLAKES | 6.8 | 1.9 | 87 | 385 | 0.928 | 0.704 |
| CEREAL DESAYUNO ARROZ CROCANTE | CEREALS RTE,KELLOGG,KELLOGG'S CORN POPS | 4.6 | 1.3 | 90.4 | 389 | 0.34 | 0.282 |
| CEREAL DESAYUNO CON PASAS | CEREALS RTE,KELLOGG,KELLOGG'S RAISIN BRAN | 7.72 | 2.72 | 77.29 | 314 | - | - |
| CEREAL DESAYUNO FROOT LOOPS, TRIX | CEREALS RTE,KELLOGG,KELLOGG'S FROOT LOOPS | 5 | 3.3 | 86.7 | 372 | - | - |
| CEREAL DESAYUNO GRANOLA CON TRIGO Y MIEL | CEREALS RTE,QUAKER,100% NAT GRANOLA,OATS,WHEAT & HONEY | 10.55 | 11.62 | 73.65 | 421 | 0.682 | 0.275 |
| CEREAL DESAYUNO HOJUELAS AZUCARADAS | CEREALS RTE,KELLOGG,KELLOGG'S FRSTD FLAKES | 3.96 | 1.68 | 89.2 | 369 | 0.52 | 0.312 |
| CEREAL DESAYUNO HOJUELAS DE TRIGO ALL-BRAN | CEREALS RTE,KELLOGG,KELLOGG'S ALL-BRAN COMPLETE WHEAT FLAKES | 10 | 2 | 79 | 318 | 0.013 | 0.01 |
| CEREAL DESAYUNO HOJUELAS SABOR A CHOCOLATE | CEREALS RTE,KELLOGG,KELLOGG'S COCOA KRISPIES | 5.22 | 2.9 | 86.05 | 389 | 0.40 | 0.33 |
| CEREAL DESAYUNO MUESLI CON FRUTAS SECAS Y NUECES | CEREALS RTE,MUESLI,DRIED FRUIT & NUTS | 9.7 | 4.9 | 77.8 | 340 | 0.40 | 0.33 |
| CEREAL DESAYUNO ORIGINAL | CEREALS RTE,KELLOGG,KELLOGG'S CORN FLAKES | 7.5 | 0.4 | 84.1 | 357 | 0.40 | 0.33 |
| CEREAL DESAYUNO SPECIAL K | CEREALS RTE,KELLOGG,KELLOGG'S SPL K | 18.7 | 1.7 | 73 | 378 | 0.40 | 0.33 |
| CHOCLO AMARILLO (EN MAZORCA) | ELOTE AMARILLO | 3.30 | 1.30 | 25.10 | 108 | 0.16 | 0.13 |
| CHOCLO BLANCO | CORN,SWEET,WHITE,RAW | 3.22 | 1.18 | 19.02 | 86 | 0.15 | 0.123 |
| FIDEO CHINO FORTIFICADO (COCIDO) | NOODLES,EGG,CKD,ENR | 4.54 | 2.07 | 25.16 | 138 | 0.154 | 0.109 |
| FIDEO CHINO FORTIFICADO (SECO) | NOODLES,EGG,DRY,ENRICHED | 14.16 | 4.44 | 71.27 | 384 | 0.59 | 0.488 |
| FIDEO DE ARROZ (COCIDO) | RICE NOODLES,COOKED | 0.91 | 0.2 | 24.9 | 109 | 0.593 | 0.267 |
| FIDEO DE ARROZ (SECO) | RICE NOODLES,DRY | 3.44 | 0.56 | 83.24 | 364 | 0.595 | 0.436 |
| FIDEO/PASTA PROMEDIO | PASTAS SIN ENRIQUECER PROMEDIO | 10.30 | 1.00 | 76.30 | 364 | 0.285 | 0.236 |
| FIDEO/SPAGHETTI DE ESPINACA (COCIDO) | SPAGHETTI,SPINACH,COOKED | 4.58 | 0.63 | 26.15 | 130 | 0.28 | 0.24 |
| FIDEO/SPAGHETTI DE ESPINACA (SECO) | SPAGHETTI,SPINACH,DRY | 13.35 | 1.57 | 74.81 | 372 | 0.681 | 0.357 |
| FIDEO/SPAGHETTI DE TRIGO ENTERO (COCIDO) | SPAGHETTI,WHOLE-WHEAT,CKD | 5.33 | 0.54 | 26.54 | 124 | - | - |
| FIDEO/SPAGHETTI DE TRIGO ENTERO (SECO) | SPAGHETTI,WHOLE-WHEAT,DRY | 14.63 | 1.4 | 75.03 | 348 | - | - |
| FIDEO/SPAGUETTI FORTIFICADO | SPAGHETTI,DRY,ENRICHED | 13.04 | 1.51 | 74.67 | 371 | 0.23 | 0.06 |
| GALLETAS COBERTURA DE CHOCOLATE | COOKIES,GRAHAM CRACKERS,CHOCOLATE-COATED | 5.8 | 23.2 | 66.5 | 484 | 0.233 | 0.138 |
| GALLETAS CON CHISPAS DE CHOCOLATE | COOKIES,CHOC CHIP,COMMLY PREP,REG,HIGHER FAT,UNENR | 5.4 | 22.6 | 66.8 | 481 | - | - |
| GALLETAS CON RELLENO TIPO WAFFER | COOKIES,SUGAR WAFERS W/CREME FILLING,REG | 3.84 | 23.24 | 70.64 | 502 | 0.737 | 0.436 |
| GALLETAS DE AVENA | COOKIES,OATMEAL,COMMLY PREP,REG | 6.2 | 18.1 | 68.7 | 450 |  |  |
| GALLETAS DE SAL TIPO SALTINAS | CRACKERS,SALTINES (INCL OYSTER,SODA,SOUP) | 9.5 | 8.85 | 74.34 | 421 | 0.374 | 0.079 |
| GALLETAS OREO | NABISCO,NABISCO OREO CRUNCHIES,COOKIE CRUMB TOPPING | 4.78 | 21.5 | 70.23 | 476 | - | - |
| GALLETAS SIMPLES DE DULCE | COOKIES,SUGAR,COMMLY PREP,REG (INCL VANILLA) | 5.1 | 21.1 | 67.9 | 478 | 0.326 | 0.154 |
| GERMEN DE TRIGO | WHEAT GERM,CRUDE | 23.15 | 9.72 | 51.8 | 360 | - | - |
| GRANOLA EN BARRA NUTRIGRAIN | KELLOGG,KELLOGG'S NUTRI-GRAIN CRL BARS,MXD BERRY | 4.3 | 7.6 | 72.8 | 370 | 0.223 | 0.093 |
| GRANOLA PREPARADA EN CASA | CEREALS RTE,GRANOLA,HOMEMADE | 14.85 | 24.06 | 53.25 | 489 | - | - |
| HARINA DE CEBADA/ MÁCHICA | HARINA CEBADA | 8.6 | 0.7 | 77.4 | 306 | 0.748 | 0.293 |
| HARINA DE CENTENO | RYE FLOUR,MEDIUM | 10.88 | 1.52 | 75.43 | 349 | 0.24 | 0.094 |
| HARINA DE MAÍZ | CORN FLR,WHOLE-GRAIN,YEL | 6.93 | 3.86 | 76.85 | 361 | 0.642 | 0.355 |
| HARINA DE QUINUA | HARINA DE QUINUA | 9.1 | 2.6 | 72.1 | 341 | 0.22 | 0.122 |
| HARINA DE TRIGO INTEGRAL | WHEAT FLOUR,WHOLE-GRAIN | 13.21 | 2.5 | 71.97 | 340 | 0.52 | 0.31 |
| HARINA DE TRIGO REFINADA Y FORTIFICADA | WHEAT FLR,WHITE,ALL-PURPOSE,ENR,BLEACHED | 10.33 | 0.98 | 76.31 | 364 | 0.728 | 0.382 |
| MAICENA | CORNSTARCH | 0.26 | 0.05 | 91.27 | 381 | 0.265 | 0.139 |
| MAÍZ (NEGRO O AZUL) | MAÍZ (NEGRO O AZUL) | 8.10 | 3.60 | 76.90 | 362 | 0.184 | 0.115 |
| MAÍZ AMARILLO | MAÍZ AMARILLO | 8.10 | 3.60 | 76.90 | 362 | 0.049 | 0.03 |
| MELVAS CHICAS |  | 5.88 | 29.41 | 52.94 | 529 | 0.668 | 0.243 |
| MOROCHO | MAÍZ BLANCO | 8.10 | 3.60 | 76.90 | 362 | 0.004 | 0.002 |
| MOTE | MAÍZ BLANCO | 8.10 | 3.60 | 76.90 | 362 | 0.45 | 0.182 |
| OREJAS (LA CANASTA Y TODAS LAS MARCAS) |  | 8.33 | 25.00 | 66.67 | 500 | 0.301 | 0.213 |
| PAN BLANCO INDUSTRIAL TIPO SUPAN | BREAD,WHITE,COMMLY PREP (INCL SOFT BREAD CRUMBS) | 9.15 | 3.19 | 49.06 | 265 | 0.261 | 0.173 |
| PAN BLANCO INDUSTRIAL TIPO SUPAN (TOSTADO) | BREAD,WHITE,COMMLY PREP,TSTD | 9 | 4 | 54.4 | 293 |  |  |
| PAN DE CENTENO | BREAD,RYE | 8.5 | 3.3 | 48.3 | 259 | 0.192 | 0.121 |
| PAN DE CENTENO (TOSTADO) | BREAD,RYE,TOASTED | 9.4 | 3.6 | 53.1 | 284 | 0.295 | 0.202 |
| PAN DE GERMEN DE TRIGO | BREAD,WHEAT GERM | 9.6 | 2.9 | 48.3 | 261 | 0.25 | 0.175 |
| PAN DE SALVADO DE TRIGO | BREAD,WHEAT BRAN | 8.8 | 3.4 | 47.8 | 248 | - | - |
| PAN DE TRIGO | BREAD,WHEAT | 10.37 | 3.44 | 49.46 | 270 | - | - |
| PAN DE TRIGO (TOSTADO) | BREAD, WHEAT, TSTD | 12.96 | 4.27 | 55.77 | 313 | 0.283 | 0.175 |
| PAN INTEGRAL DE TRIGO | BREAD,WHOLE-WHEAT,COMM. PREPARED | 12.95 | 3.35 | 41.29 | 247 | 0.308 | 0.191 |
| PAN INTEGRAL DE TRIGO (TOSTADO) | BREAD, WHOLE-WHEAT, COMMER PREP, TOASTED | 16.27 | 4.07 | 51.16 | 306 | - | - |
| PAN NEGRO DE CENTENO | BREAD,PUMPERNICKEL | 8.7 | 3.1 | 47.5 | 250 | 0.444 | 0.261 |
| PAN NEGRO DE CENTENO (TOSTADO) | BREAD,PUMPERNICKEL,TSTD | 9.5 | 3.4 | 52.2 | 275 | 0.446 | 0.257 |
| PAN PARA HAMBURGUESA O HOTDOG MULTICEREAL | ROLLS,HAMBURGER OR HOTDOG,MIXED-GRAIN | 9.6 | 6 | 44.6 | 263 | 0.47 | 0.292 |
| PAN PARA HAMBURGUESA O HOTDOG REGULAR | ROLLS,HAMBURGER OR HOTDOG,PLN | 9.87 | 3.76 | 50.15 | 278 | 0.459 | 0.264 |
| PAN PITA BLANCO | BREAD,PITA,WHITE,ENRICHED | 9.1 | 1.2 | 55.7 | 275 | - | - |
| PAN PITA INTEGRAL | BREAD,PITA,WHOLE-WHEAT | 9.8 | 2.6 | 55 | 266 | 0.411 | 0.213 |
| QUINUA | QUINOA,UNCKD | 14.12 | 6.07 | 64.16 | 368 | 0.451 | 0.235 |
| SALVADO DE TRIGO | WHEAT BRAN,CRUDE | 15.55 | 4.25 | 64.51 | 216 | - | - |
| SEMOLA | SEMOLINA,UNENRICHED | 12.68 | 1.05 | 72.83 | 360 | 0.315 | 0.165 |
| TAPIOCA | TAPIOCA,PEARL,DRY | 0.19 | 0.02 | 88.69 | 358 | 0.419 | 0.252 |
| TORTILLA DE MAÍZ AMARILLO | TORTILLAS,RTB OR -FRY,CORN | 5.7 | 2.85 | 44.64 | 218 | 0.458 | 0.293 |
| TORTILLA DE MAÍZ Y TRIGO | TORTILLA DE MAÍZ Y TRIGO | 5.70 | 2.50 | 46.60 | 222 | 0.422 | 0.238 |
| TRIGO ENTERO | WHEAT,DURUM | 13.68 | 2.47 | 71.13 | 339 | 0.464 | 0.261 |
|  | |  |  |  |  |  |  |
| ACEITUNAS | OLIVES,RIPE,CND (SMALL-EXTRA LRG) | 0.84 | 10.68 | 6.26 | 115 | 1.141 | 0.594 |
| AJONJOLI | SESAME SEEDS,WHOLE,DRIED | 17.73 | 49.67 | 23.45 | 573 | 0.485 | 0.253 |
| ALMENDRA | ALMONDS | 21.22 | 49.42 | 21.67 | 575 | 1.275 | 0.664 |
| ARVEJA | PEAS,GREEN,RAW | 5.42 | 0.4 | 14.45 | 81 | 0.511 | 0.205 |
| ARVEJA (ENLATADA) | PEAS,GRN,CND,REG PK,SOL&LIQUIDS | 3.01 | 0.48 | 10.6 | 58 | 0.309 | 0.124 |
| ARVEJA COCIDA EN AGUA SIN SAL | PEAS,GRN,CKD,BLD,DRND,WO/SALT | 5.36 | 0.22 | 15.63 | 84 | 1.168 | 0.608 |
| ARVEJA CONGELADA Y COCIDA SIN SAL (COMERCIAL) | PEAS,GRN,FRZ,CKD,BLD,DRND,WO/SALT | 5.15 | 0.27 | 14.26 | 78 | 0.479 | 0.25 |
| AVELLANA | HAZELNUTS OR FILBERTS | 14.95 | 60.75 | 16.7 | 628 | 1.133 | 0.59 |
| CASTAÑA | CHESTNUTS,CHINESE,RAW | 4.2 | 1.11 | 49.07 | 224 | 0.49 | 0.255 |
| CHIA | CHIA SEEDS,DRIED | 16.54 | 30.74 | 42.12 | 486 | 1.182 | 0.615 |
| CHOCHO | LUPINS,MATURE SEEDS,RAW | 36.17 | 9.74 | 40.37 | 371 | 1.095 | 0.427 |
| CHOCHO COCIDO EN AGUA CON SAL | LUPINS,MATURE SEEDS,CKD,BLD,W/SALT | 15.57 | 2.92 | 9.29 | 116 | 0.228 | 0.196 |
| CHOCHO COCIDO EN AGUA SIN SAL | LUPINS,MATURE SEEDS,CKD,BLD,WO/ SALT | 15.57 | 2.92 | 9.88 | 119 | 1.103 | 0.827 |
| FRÉJOL BLANCO | BEANS,SML WHITE,MATURE SEEDS,RAW | 21.11 | 1.18 | 62.25 | 336 | 0.2 | 0.114 |
| FRÉJOL BLANCO COCIDO EN AGUA SIN SAL | BEANS,SML WHITE,MATURE SEEDS,CKD,BLD,WO/SALT | 8.97 | 0.64 | 25.81 | 142 | 0.198 | 0.112 |
| FREJOL CON CARNE MOLIDA DE RES ENLATADA | CHILI WITH BEANS,CANNED | 5.71 | 5.49 | 11.91 | 112 | 0.114 | 0.065 |
| FRÉJOL DE ARBOL (GANDUL) | BEANS,GREAT NORTHERN,MATURE SEEDS,RAW | 21.86 | 1.14 | 62.37 | 339 | 0.19 | 0.108 |
| FRÉJOL GARBANCILLO | BEANS,PINTO,MATURE SEEDS,RAW | 21.42 | 1.23 | 62.55 | 347 | 1.435 | 1.36 |
| FRÉJOL NEGRO | BEANS,BLACK,MATURE SEEDS,RAW | 21.6 | 1.42 | 62.36 | 341 | 0.618 | 0.585 |
| FRÉJOL NEGRO (ENLATADO) | BEANS,BLACK TURTLE,MATURE SEEDS,CND | 6.03 | 0.29 | 16.56 | 91 | 0.618 | 0.585 |
| FRÉJOL NEGRO COCIDO EN AGUA SIN SAL | BEANS,BLACK,MATURE SEEDS,CKD,BLD,WO/SALT | 8.86 | 0.54 | 23.71 | 132 | 1.034 | 0.479 |
| FRÉJOL ROJO | BEANS,KIDNEY,ALL TYPES,MATURE SEEDS,RAW | 23.58 | 0.83 | 60.01 | 333 | 0.475 | 0.22 |
| FRÉJOL ROJO (ENLATADO) | BEANS,KIDNEY,ALL TYPES,MATURE SEEDS,CND | 5.22 | 0.6 | 14.5 | 82 | 0.265 | 0.123 |
| FRÉJOL ROJO COCIDO EN AGUA SIN SAL | BEANS,KIDNEY,ALL TYPES,MATURE SEEDS,CKD,BLD,WO/SALT | 8.67 | 0.5 | 22.8 | 127 | - | - |
| FRÉJOL ROSADO | BEANS,PINK,MATURE SEEDS,RAW | 20.96 | 1.13 | 64.19 | 343 | - | - |
| FRÉJOL ROSADO COCIDO EN AGUA SIN SAL | BEANS,PINK,MATURE SEEDS,CKD,BLD,WO/SALT | 9.06 | 0.49 | 27.91 | 149 | - | - |
| GARBANZO | CHICKPEAS (GARBANZO BNS,BENGAL GM),MATURE SEEDS,RAW | 19.3 | 6.04 | 60.65 | 364 | 1.273 | 0.689 |
| GARBANZO (ENLATADO) | CHICKPEAS (GARBANZO BNS,BENGAL GRAM),MATURE SDS,CND,SOL&LIQ | 4.92 | 1.95 | 13.49 | 88 | 0.445 | 0.241 |
| GARBANZO (HARINA) | CHICKPEA FLOUR (BESAN) | 22.39 | 6.69 | 57.82 | 387 | 0.442 | 0.252 |
| GARBANZO COCIDO EN AGUA SIN SAL | CHICKPEAS ,MATURE SEEDS,CKD,BLD,WO/SALT | 8.86 | 2.59 | 27.42 | 164 | 0.434 | 0.248 |
| HABA | BROADBEANS (FAVA BEANS),MATURE SEEDS,RAW | 26.12 | 1.53 | 58.29 | 341 | 0.292 | 0.164 |
| HABA (HARINA) | HARINA HABA | 24.3 | 1.9 | 59.6 | 343 | 0.09 | 0.074 |
| HABA TIERNA | BROADBEANS,IMMAT SEEDS,RAW | 5.6 | 0.6 | 11.7 | 72 | 0.326 | 0.17 |
| HUMMUS COMERCIAL | HUMMUS,COMMERCIAL | 7.9 | 9.6 | 14.29 | 166 | 2.122 | 1.539 |
| LENTEJA | LENTILS,RAW | 25.8 | 1.06 | 60.08 | 353 | 0.869 | 0.63 |
| LENTEJA COCIDA EN AGUA SIN SAL | LENTILS,MATURE SEEDS,CKD,BLD,WO/SALT | 9.02 | 0.38 | 20.13 | 116 | 0.486 | 0.352 |
| LENTEJA GERMINADA | LENTILS,SPROUTED,RAW | 8.96 | 0.55 | 22.14 | 106 | - | - |
| LENTEJA GERMINADA COCIDA EN AGUA SIN SAL | LENTILS,SPROUTED,CKD,STIR-FRIED,WO/SALT | 8.8 | 0.45 | 21.25 | 101 | - | - |
| LINAZA | SEEDS,FLAXSEED | 18.29 | 42.16 | 28.88 | 534 | 0.029 | 0.023 |
| MACADAMIA | MACADAMIA NUTS,RAW | 7.91 | 75.77 | 13.82 | 718 | 0.94 | 0.743 |
| MAÍZ DULCE (ENLATADO) | CORN,SWT,YEL,CND,BRINE PK,REG PK,SOL&LIQUIDS | 1.95 | 0.77 | 13.86 | 61 | 1.12 | 0.452 |
| MANI | PEANUTS,ALL TYPES,RAW | 25.8 | 49.24 | 16.13 | 567 | 0.663 | 0.362 |
| MANI COCIDO CON SAL | PEANUTS,ALL TYPES,CKD,BLD,W/SALT | 13.5 | 22.01 | 21.26 | 318 | 0.19 | 0.125 |
| MANI TOSTADO CON ACEITE Y SAL | PEANUTS,ALL TYPES,OIL-ROASTED,W/SALT | 28.03 | 52.5 | 15.26 | 599 | 1.169 | 0.666 |
| MANI TOSTADO CON SAL | PEANUTS,ALL TYPES,DRY-ROASTED,W/SALT | 23.68 | 49.66 | 21.51 | 585 | 0.957 | 0.493 |
| MANTEQUILLA DE MANÍ BAJA EN GRASA | PEANUT BUTTER,SMOOTH,RED FAT | 25.9 | 34 | 35.65 | 520 | 1.016 | 0.563 |
| MANTEQUILLA DE MANÍ CON SAL | PEANUT BUTTER,SMOOTH STYLE,W/ SALT | 25.09 | 50.39 | 19.56 | 588 | 1.337 | 1.049 |
| MISO | MISO | 11.69 | 6.01 | 26.47 | 199 | 0.7 | 0.549 |
| NUEZ | WALNUTS,ENGLISH | 15.23 | 65.21 | 13.71 | 654 | 1.427 | 1.006 |
| PEPAS DE ZAMBO | PUMPKIN&SQUASH SEEDS,WHL,RSTD,WO/SALT | 18.55 | 19.4 | 53.75 | 446 | 1.227 | 0.963 |
| PISTACHO | PISTACHIO NUTS,DRY RSTD,WO/SALT | 20.95 | 44.82 | 29.38 | 567 | 0.711 | 0.406 |
| SEMILLAS DE GIRASOL | SUNFLOWER SD KRNLS,DRIED | 20.78 | 51.46 | 20 | 584 | 1.18 | 0.814 |
| SOYA | SOYBEANS,MATURE SEEDS,RAW | 36.49 | 19.94 | 30.16 | 446 |  |  |
| SOYA (LECHE EN POLVO) |  | 24.24 | 3.03 | 69.70 | 424 | 1.107 | 0.507 |
| SOYA (LECHE) | SOYMILK (ALL FLAVORS),LOWFAT,W/ ADDED CA,VITAMINS A & D | 1.65 | 0.62 | 7.2 | 43 | 0.924 | 0.77 |
| SOYA COCIDA EN AGUA SIN SAL | SOYBEANS,MATURE CKD,BLD,WO/SALT | 16.64 | 8.97 | 9.93 | 173 | 0.665 | 0.511 |
|  | |  |  |  |  |  |  |
| ACELGA | CHARD,SWISS,RAW | 1.8 | 0.2 | 3.74 | 19 | 0.11 | - |
| ACELGA COCIDA SIN SAL | CHARD,SWISS,CKD,BLD,DRND,WO/SALT | 1.88 | 0.08 | 4.13 | 20 | 0.114 | - |
| ACHOJCHA | BALSAM-PEAR (BITTER GOURD),PODS,RAW | 1 | 0.17 | 3.7 | 17 | - | - |
| AJÍ ROCOTO ROJO | PEPPERS,HOT CHILI,RED,RAW | 1.87 | 0.44 | 8.81 | 40 | 0.062 | 0.042 |
| AJO | GARLIC,RAW | 6.36 | 0.5 | 33.06 | 149 | 0.183 | 0.081 |
| ALBAHACA | BASIL,FRESH | 3.15 | 0.64 | 2.65 | 23 | 0.13 | 0.077 |
| ALCACHOFA | ARTICHOKES,(GLOBE OR FRENCH),RAW | 3.27 | 0.15 | 10.51 | 47 | - | - |
| ALCACHOFA COCIDA SIN SAL | ARTICHOKES,(GLOBE OR FRENCH),CKD,BLD,DRND,WO/SALT | 2.89 | 0.34 | 11.95 | 53 | - | - |
| ALMIDÓN DE ACHIRA | ARROWROOT FLOUR | 0.3 | 0.1 | 88.15 | 357 | 0.012 | 0.009 |
| ALMIDÓN DE YUCA | FÉCULA MANDIOCA | 0.5 | 0.3 | 81.1 | 331 | - | - |
| APIO | CELERY,RAW | 0.69 | 0.17 | 2.97 | 16 | 0.02 | 0.009 |
| BERENJENA | EGGPLANT,RAW | 0.98 | 0.18 | 5.88 | 25 | 0.043 | 0.027 |
| BERENJENA COCIDA SIN SAL | EGGPLANT,CKD,BLD,DRND,WO/SALT | 0.83 | 0.23 | 8.73 | 35 | 0.035 | 0.022 |
| BERRO | WATERCRESS,RAW | 2.3 | 0.1 | 1.29 | 11 | 0.114 | 0.063 |
| BRÓCOLI | BROCCOLI,RAW | 2.82 | 0.37 | 6.64 | 34 | 0.117 | 0.05 |
| BRÓCOLI COCIDO SIN SAL | BROCCOLI,CKD,BLD,DRND,WO/SALT | 2.38 | 0.41 | 7.18 | 35 | 0.116 | 0.06 |
| CAMOTE | SWEET POTATO,RAW,UNPREP | 1.57 | 0.05 | 20.12 | 86 | 0.089 | 0.034 |
| CAMOTE COCIDO | SWEET POTATO,CKD,BLD,WO/ SKN | 1.37 | 0.14 | 17.72 | 76 | 0.078 | 0.03 |
| CEBOLLA BLANCA | ONIONS,SPRING OR SCALLIONS (INCL TOPS&BULB),RAW | 1.83 | 0.19 | 7.34 | 32 | 0.059 | 0.053 |
| CEBOLLA PAITEÑA | ONIONS,RAW | 1.1 | 0.1 | 9.34 | 40 | 0.025 | 0.014 |
| CEBOLLA PERLA | ONIONS,RAW | 1.1 | 0.1 | 9.34 | 40 | 0.025 | 0.014 |
| CEBOLLA PUERRO | LEEKS,(BULB&LOWER LEAF-PORTION),RAW | 1.5 | 0.3 | 14.15 | 61 | 0.055 | 0.041 |
| CEBOLLIN | CHIVES,RAW | 3.27 | 0.73 | 4.35 | 30 | 0.105 | 0.095 |
| CHAMPIÑONES | MUSHROOMS,WHITE,RAW | 3.09 | 0.34 | 3.26 | 22 | 0.085 | 0.044 |
| CHAMPIÑONES (ENLATADOS) | MUSHROOMS,CND,DRND SOL | 1.87 | 0.29 | 5.09 | 25 | 0.052 | 0.026 |
| CHAMPIÑONES COCIDOS SIN SAL | MUSHROOMS,WHITE,CKD,BLD,DRND,WO/ SALT | 2.17 | 0.47 | 5.29 | 28 | 0.06 | 0.031 |
| CHAMPIÑONES PORTOBELLO | MUSHROOMS,PORTABELLA,RAW | 2.11 | 0.35 | 3.87 | 22 | 0.076 | 0.014 |
| CILANTRO | CORIANDER (CILANTRO) LEAVES,RAW | 2.13 | 0.52 | 3.67 | 23 | - | - |
| COL BLANCA | CABBAGE,RAW | 1.28 | 0.1 | 5.8 | 25 | 0.032 | 0.019 |
| COL BLANCA COCIDA SIN SAL | CABBAGE,CKD,BLD,DRND,WO/SALT | 1.27 | 0.06 | 5.51 | 23 | 0.032 | 0.019 |
| COL DE BRUSELAS | BRUSSELS SPROUTS,RAW | 3.38 | 0.3 | 8.95 | 43 | 0.098 | - |
| COL DE BRUSELAS COCIDA SIN SAL | BRUSSELS SPROUTS,CKD,BLD,DRND,WO/SALT | 2.55 | 0.5 | 7.1 | 36 | 0.074 | - |
| COL MORADA | CABBAGE,RED,RAW | 1.43 | 0.16 | 7.37 | 31 | 0.036 | 0.022 |
| COL MORADA COCIDA SIN SAL | CABBAGE,RED,CKD,BLD,DRND,WO/SALT | 1.51 | 0.09 | 6.94 | 29 | 0.038 | 0.023 |
| COLIFLOR | CAULIFLOWER,RAW | 1.92 | 0.28 | 4.97 | 25 | 0.065 | 0.051 |
| COLIFLOR COCIDO SIN SAL | CAULIFLOWER,CKD,BLD,DRND,WO/SALT | 1.84 | 0.45 | 4.11 | 23 | 0.066 | 0.04 |
| ESPÁRRAGO | ASPARAGUS,RAW | 2.2 | 0.12 | 3.88 | 20 | 0.075 | 0.052 |
| ESPÁRRAGO (ENLATADO) | ASPARAGUS,CND,REG PK,SOL&LIQUIDS | 1.8 | 0.18 | 2.47 | 15 | 0.043 | 0.029 |
| ESPÁRRAGO COCIDO | ASPARAGUS,CKD,BLD,DRND | 2.4 | 0.22 | 4.11 | 22 | 0.082 | 0.057 |
| ESPINACA | SPINACH,RAW | 2.86 | 0.39 | 3.63 | 23 | 0.129 | 0.108 |
| ESPINACA COCIDA SIN SAL | SPINACH,CKD,BLD,DRND,WO/ SALT | 2.97 | 0.26 | 3.75 | 23 | 0.134 | 0.113 |
| LECHUGA | LETTUCE,GRN LEAF,RAW | 1.36 | 0.15 | 2.87 | 15 | 0.055 | 0.032 |
| LECHUGA CRESPA | LETTUCE,COS OR ROMAINE,RAW | 1.23 | 0.3 | 3.29 | 17 | 0.065 | 0.025 |
| MELLOCO | MELLOCO | 1.1 | 0.1 | 14.3 | 62 | - | - |
| NABO | TURNIPS,RAW | 0.9 | 0.1 | 6.43 | 28 | 0.017 | 0.013 |
| NABO COCIDO SIN SAL | TURNIPS,CKD,BLD,DRND,WO/SALT | 0.71 | 0.08 | 5.06 | 22 | 0.014 | 0.011 |
| ÑAME | YAM,RAW | 1.53 | 0.17 | 27.88 | 118 | 0.071 | 0.04 |
| OCA | OCA | 0.8 | 0.4 | 15.4 | 30 | - | - |
| PALMITO | PALM HEARTS,RAW | 2.7 | 0.2 | 25.61 | 115 | - | - |
| PALMITO (ENLATADO) | HEARTS OF PALM,CANNED | 2.52 | 0.62 | 4.62 | 28 | 0.098 | 0.049 |
| PAN DE YUCA CRUDO | PãO, DE QUEIJO, CRU | 3.6 | 14.0 | 38.5 | 295 | - | - |
| PAPA AMARILLA | PAPA AMARILLA | 2 | 0.4 | 23.3 | 103 | - | - |
| PAPA NABO | TURNIPS,RAW | 0.9 | 0.1 | 6.43 | 28 | 0.017 | 0.013 |
| PAPA NABO COCIDO SIN SAL | TURNIPS,CKD,BLD,DRND,WO/SALT | 0.71 | 0.08 | 5.06 | 22 | 0.014 | 0.011 |
| PAPA PROMEDIO | PAPA PROMEDIO | 2.1 | 0.1 | 22.3 | 97 | - | - |
| PEPINILLO | CUCUMBER,WITH PEEL,RAW | 0.65 | 0.11 | 3.63 | 15 | 0.019 | 0.011 |
| PEREJIL | PARSLEY,FRSH | 2.97 | 0.79 | 6.33 | 36 | 0.145 | 0.082 |
| PIMIENTO ROJO | PEPPERS,SWT,RED,RAW | 0.99 | 0.3 | 6.03 | 31 | 0.05 | 0.009 |
| PIMIENTO VERDE | PEPPERS,SWT,GRN,RAW | 0.86 | 0.17 | 4.64 | 20 | 0.092 | 0.012 |
| PLÁTANO (HARINA) | HARINA DE PLÁTANO | 3.1 | 0.4 | 79.6 | 300 | - | - |
| PLÁTANO DE SEDA (BANANO/GUINEO) | BANANAS,RAW | 1.09 | 0.33 | 22.84 | 89 | 0.049 | 0.009 |
| PLÁTANO MADURO/MAQUEÑO | PLÁTANO MADURO | 1.2 | 0.2 | 27 | 112 | - | - |
| PLÁTANO ORITO | PLATANO DOMINICO | 1.10 | 0.20 | 27.40 | 104 | - | - |
| PLÁTANO ROSADO | PLATANO MORADO | 0.60 | 0.10 | 23.10 | 86 | 0.02 | 0.01 |
| PLÁTANO VERDE | PLANTAINS,RAW | 1.3 | 0.37 | 31.89 | 122 | 0.044 | 0.032 |
| PLÁTANO VERDE COCIDO | PLANTAINS,COOKED | 0.79 | 0.18 | 31.15 | 116 | 0.027 | 0.02 |
| RÁBANO | RADISHES,RAW | 0.68 | 0.1 | 3.4 | 16 | 0.036 | 0.009 |
| RÁBANO LARGO | RADISHES,ORIENTAL,RAW | 0.6 | 0.1 | 4.1 | 18 | 0.02 | 0.011 |
| REMOLACHA | BEETS,RAW | 1.61 | 0.17 | 9.56 | 43 | 0.046 | 0.038 |
| REMOLACHA COCIDA | BEETS,CKD,BLD,DRND | 1.68 | 0.18 | 9.96 | 44 | 0.048 | 0.04 |
| RÚCULA | ARUGULA,RAW | 2.58 | 0.66 | 3.65 | 25 | - | - |
| SAMBO | GOURD,WHITE-FLOWERED (CALABASH),RAW | 0.62 | 0.02 | 3.39 | 14 | 0.015 | - |
| SAMBO COCIDO SIN SAL | GOURD,WHITE-FLOWERED (CALABASH),CKD,BLD,DRND,WO/SALT | 0.6 | 0.02 | 3.69 | 15 | 0.014 | - |
| TOMATE RIÑON | TOMATOES,RED,RIPE,RAW,YEAR RND AVERAGE | 0.88 | 0.2 | 3.89 | 18 | 0.027 | 0.014 |
| VAINITAS | BEANS,SNAP,GREEN,RAW | 1.83 | 0.22 | 6.97 | 31 | 0.067 | 0.042 |
| VAINITAS COCIDAS SIN SAL | BEANS,SNAP,GRN,CKD,BLD,DRND,WO/SALT | 1.89 | 0.28 | 7.88 | 35 | 0.069 | 0.044 |
| YERBABUENA | SPEARMINT,FRESH | 3.29 | 0.73 | 8.41 | 44 | 0.168 | 0.099 |
| YUCA | CASSAVA,RAW | 1.36 | 0.28 | 38.06 | 160 | 0.026 | 0.017 |
| ZANAHORIA | CARROTS,RAW | 0.93 | 0.24 | 9.58 | 41 | 0.061 | 0.043 |
| ZANAHORIA (ZUMO) | ZANAHORIA (JUGO) | 1.10 | 0.20 | 10.50 | 45 | 0.03 | 0.02 |
| ZANAHORIA BLANCA | PARSNIPS,RAW | 1.2 | 0.3 | 17.99 | 75 | - | - |
| ZANAHORIA BLANCA COCIDA SIN SAL | PARSNIPS,CKD,BLD,DRND,WO/SALT | 1.32 | 0.3 | 17.01 | 71 | - | - |
| ZANAHORIA COCIDA SIN SAL | CARROTS,CKD,BLD,DRND,WO/SALT | 0.76 | 0.18 | 8.22 | 35 | 0.05 | 0.035 |
| ZAPALLO | SQUASH,WINTER,HUBBARD,RAW | 2 | 0.5 | 8.7 | 40 | 0.078 | 0.067 |
| ZAPALLO COCIDO | SQUASH,WNTR,HUBBARD,BKD,WO/ SALT | 2.48 | 0.62 | 10.81 | 50 | 0.058 | 0.05 |
| ZUQUINI | SQUASH,SMMR,ZUCCHINI,INCL SKN,RAW | 1.21 | 0.32 | 3.11 | 17 | 0.043 | 0.032 |
| ZUQUINI COCIDO SIN SAL | SQUASH,SMMR,ZUCCHINI,INCL SKN,CKD,BLD,DRND,WO/SALT | 1.14 | 0.36 | 2.69 | 15 | 0.022 | 0.017 |
|  | |  |  |  |  |  |  |
| AGUACATE (PROMEDIO) | AVOCADOS,RAW,ALL COMM VAR | 2 | 14.66 | 8.53 | 160 | 0.097 | 0.049 |
| AGUACATE SERRANO | AVOCADOS,RAW,FLORIDA | 2.23 | 10.06 | 7.82 | 120 | 0.108 | 0.054 |
| CACAO SECO | CACAO CON CASCARA | 12.00 | 46.30 | 34.70 | 456 | - | - |
| CAÑA DE AZUCAR | CAÑA DE AZUCAR | 0.00 | 0.00 | 6.80 | 26 | 0.037 | 0.044 |
| CAÑA DE AZÚCAR (JUGO) | CAÑA DE AZÚCAR (JUGO) | 0.30 | 0.10 | 20.50 | 82 | - | - |
| CAPULI | CAPULIN | 2.10 | 2.30 | 17.90 | 91 | - | - |
| CARAMBOLA | CARAMBOLA,(STARFRUIT),RAW | 1.04 | 0.33 | 6.73 | 31 | - | - |
| CEREZA | CHERRIES,SOUR,RED,RAW | 1 | 0.3 | 12.18 | 50 | 0.042 | 0.031 |
| CEREZAS EN CONSERVA | CHERRIES,SWT,CND,LT SYRUP PK,SOL&LIQUIDS | 0.61 | 0.15 | 17.29 | 67 | 0.014 | 0.008 |
| CHIRIMOYA | CHERIMOYA,RAW | 1.57 | 0.68 | 17.71 | 75 | - | - |
| CHONTADURO PIJUAYO | CHONTADURO PIJUAYO | 2.8 | 3.2 | 41 | 184 | - | - |
| CIRUELA PASA | PRUNES,DEHYD (LOW-MOISTURE),UNCKD | 3.7 | 0.73 | 89.07 | 339 | 0.014 | 0.008 |
| CLAUDIA AMARILLA | PLUMS,RAW | 0.7 | 0.28 | 11.42 | 46 | 0.037 | 0.022 |
| CLAUDIA ROJA | PLUMS,RAW | 0.7 | 0.28 | 11.42 | 46 | 0.116 | 0.071 |
| COCO (AGUA) | COCONUT H2O (LIQ FROM COCONUTS) | 0.72 | 0.2 | 3.71 | 19 | 0.169 | 0.103 |
| COCO (JUGO CON PULPA) | COCONUT MILK,RAW (LIQ EXPRESSED FROM GRATED MEAT&H2O) | 2.29 | 23.84 | 5.54 | 230 | 0.349 | 0.213 |
| COCO (PULPA) | COCONUT MEAT,RAW | 3.33 | 33.49 | 15.23 | 354 | - | - |
| COCO DESHIDRATADO | COCONUT MEAT,DRIED (DESICCATED),NOT SWTND | 6.88 | 64.53 | 23.65 | 660 | 0.019 | 0.014 |
| COKTEL DE FRUTAS EN CONSERVA | FRUIT COCKTAIL,CND,EX HVY SYRUP,SOL&LIQUIDS | 0.39 | 0.07 | 22.89 | 88 | 0.015 | 0.012 |
| DURAZNO | PEACHES,RAW | 0.91 | 0.25 | 9.54 | 39 | 0.019 | 0.022 |
| DURAZNOS EN ALMIBAR | PEACHES,CND,EX HVY SYRUP PK,SOL&LIQUIDS | 0.47 | 0.03 | 26.06 | 96 | 0.019 | 0.014 |
| FRUTA CRIOLLA | PEACHES,RAW | 0.91 | 0.25 | 9.54 | 39 | 0.026 | 0.019 |
| FRUTA DE PAN | BREADFRUIT,RAW | 1.07 | 0.23 | 27.12 | 103 | - | - |
| FRUTILLA | STRAWBERRIES,RAW | 0.67 | 0.3 | 7.68 | 32 | - | - |
| GRANADILLA | PASSION-FRUIT,(GRANADILLA),PURPLE,RAW | 2.2 | 0.7 | 23.38 | 97 | 0.006 | 0.031 |
| GROSELLA | PITANGA,(SURINAM-CHERRY),RAW | 0.8 | 0.4 | 7.49 | 33 | 0.019 | 0.014 |
| GUABA | JINICUIL CRUDO (SEMILLAS) | 11.88 | 0.94 | 27.03 | 159 | 0.018 | 0.032 |
| GUANABANA | SOURSOP,RAW | 1 | 0.3 | 16.84 | 66 | 0.009 | 0.016 |
| GUAYABA | GUAVAS,COMMON,RAW | 2.55 | 0.95 | 14.32 | 68 | - | - |
| GUAYTAMBO | PEACHES,RAW | 0.91 | 0.25 | 9.54 | 39 | 0.017 | 0.012 |
| HIGOS | FIGS,RAW | 0.75 | 0.3 | 19.18 | 74 | 0.044 | 0.034 |
| HIGOS CON MIEL (CONSERVA) | FIGS,CND,EX HVY SYRUP PK,SOL&LIQUIDS | 0.38 | 0.1 | 27.86 | 107 | - | - |
| JÍCAMA | YAMBEAN (JICAMA),RAW | 0.72 | 0.09 | 8.82 | 38 | - | - |
| KIWI | KIWIFRUIT,GRN,RAW | 1.14 | 0.52 | 14.66 | 61 | - | - |
| LIMA | LIMES,RAW | 0.7 | 0.2 | 10.54 | 30 | 0.018 | 0.015 |
| LIMÓN | LEMONS,RAW,WITHOUT PEEL | 1.1 | 0.3 | 9.32 | 29 | 0.027 | 0.016 |
| LIMÓN (ZUMO) | LEMON JUICE,RAW | 0.35 | 0.24 | 6.9 | 22 | 0.006 | 0.001 |
| MANDARINA | TANGERINES,(MANDARIN ORANGES),RAW | 0.81 | 0.31 | 13.34 | 53 | - | - |
| MANGO | MANGOS,RAW | 0.82 | 0.38 | 14.98 | 60 | 0.019 | 0.014 |
| MANZANA | APPLES,RAW,WITH SKIN | 0.26 | 0.17 | 13.81 | 52 | 0.023 | 0.014 |
| MARACUYÁ | MARACUJA, CRU | 2.0 | 2.1 | 12.3 | 68 | 0.015 | 0.01 |
| MELOCOTÓN | PEACHES,RAW | 0.91 | 0.25 | 9.54 | 39 | - | - |
| MELÓN | MELONS,CANTALOUPE,RAW | 0.84 | 0.19 | 8.16 | 34 | 0.009 | 0.004 |
| MELÓN VALENCIANO | MELONS,HONEYDEW,RAW | 0.54 | 0.14 | 9.09 | 36 | 0.031 | 0.016 |
| MEMBRILLO | QUINCES,RAW | 0.4 | 0.1 | 15.3 | 57 | - | - |
| MORA | BLACKBERRIES,RAW | 1.39 | 0.49 | 9.61 | 43 | 0.021 | 0.007 |
| NARANJA | ORANGES,RAW,ALL COMM VAR | 0.94 | 0.12 | 11.75 | 47 | 0.014 | 0.013 |
| NARANJA (ZUMO) | ORANGE JUICE,RAW | 0.7 | 0.2 | 10.4 | 45 | 0.009 | 0.005 |
| NARANJILLA (PULPA CONGELADA) | NARANJILLA (LULO) PULP,FRZ,UNSWTND | 0.44 | 0.22 | 5.9 | 25 | - | - |
| NECTARINO | NECTARINES,RAW | 1.06 | 0.32 | 10.55 | 44 | 0.065 | 0.012 |
| NISPERO | LOQUATS,RAW | 0.43 | 0.2 | 12.14 | 47 | - | - |
| PAPAYA | PAPAYAS,RAW | 0.47 | 0.26 | 10.82 | 43 | - | - |
| PASAS RUBIAS | RAISINS,GOLDEN SEEDLESS | 3.39 | 0.46 | 79.52 | 302 | 0.011 | 0.002 |
| PASAS SIN SEMILLA | RAISINS,SEEDLESS | 3.07 | 0.46 | 79.18 | 299 | - | - |
| PATASMUYO O PATASMUYO | CACAU, CRU | 1.0 | 0.1 | 19.4 | 74 | 0.021 | 0.019 |
| PEPINO DULCE | PEPINO DULCE | 0.3 |  | 7 | 26 | 0.009 | 0.008 |
| PERA | PEARS,RAW | 0.36 | 0.14 | 15.23 | 57 | - | - |
| PIÑA | PINEAPPLE,RAW,ALL VAR | 0.54 | 0.12 | 13.12 | 50 | 0.015 | 0.012 |
| PIÑA EN ALMIBAR (CONSERVA) | PINEAPPLE,CND,HVY SYRUP PK,SOL&LIQUIDS | 0.35 | 0.11 | 20.2 | 78 | - | - |
| POMAROSA | ROSE-APPLES,RAW | 0.6 | 0.3 | 5.7 | 25 | - | - |
| SANDIA | WATERMELON,RAW | 0.61 | 0.15 | 7.55 | 30 | 0.041 | 0.007 |
| TAMARINDO | TAMARINDS,RAW | 2.8 | 0.6 | 62.5 | 239 | - | - |
| TOMATE DE ÁRBOL | TOMATE DE ÁRBOL | 1.3 | 0.3 | 9.8 | 41 | - | - |
| TORONJA | GRAPEFRUIT,RAW,WHITE,ALL AREAS | 0.69 | 0.1 | 8.41 | 33 | 0.038 | 0.006 |
| TUNA | PRICKLY PEARS,RAW | 0.73 | 0.51 | 9.57 | 41 | 0.041 | 0.007 |
| UVA | GRAPEFRUIT,RAW,PINK&RED&WHITE,ALL AREAS | 0.63 | 0.1 | 8.08 | 32 | - | - |
| UVA VERDE | GRAPEFRUIT,RAW,WHITE,ALL AREAS | 0.69 | 0.1 | 8.41 | 33 | - | - |
| UVILLA | GOOSEBERRIES,RAW | 0.88 | 0.58 | 10.18 | 44 | 0.061 | 0.049 |
| ZAPOTE /MAMEY | SAPOTE,MAMEY,RAW | 1.45 | 0.46 | 32.1 | 124 | - | - |
|  | |  |  |  |  |  |  |
| CLARA DE HUEVO | EGG,WHITE,RAW,FRESH | 10.9 | 0.17 | 0.73 | 52 | 0.686 | 0.457 |
| HUEVO DE CAMPO | EGG,WHL,RAW,FRSH | 12.56 | 9.51 | 0.72 | 143 | 0.681 | 0.678 |
| HUEVO DE CODORNIZ | EGG,QUAIL,WHOLE,FRESH,RAW | 13.05 | 11.09 | 0.41 | 158 | 0.68 | 0.499 |
| HUEVO ENTERO FRESCO | EGG,WHL,RAW,FRSH | 12.56 | 9.51 | 0.72 | 143 | 0.68 | 0.499 |
| YEMA DE HUEVO | EGG,YOLK,RAW,FRSH | 15.86 | 26.54 | 3.59 | 322 | 0.737 | 0.543 |
|  | |  |  |  |  |  |  |
| ALBACORA | SWORDFISH,RAW | 19.66 | 6.65 | 0 | 144 | 1.092 | 1.121 |
| ALMEJAS | CLAM,MIXED SPECIES,RAW | 14.67 | 0.96 | 3.57 | 86 | 0.773 | 0.668 |
| ATÚN ENLATADO EN AGUA SIN SAL | TUNA,LT,CND IN H2O,WO/SALT,DRND SOL | 25.51 | 0.82 | 0 | 116 | 0.56 | 0.597 |
| ATÚN ENLATADO EN ACEITE | TUNA,LT,CND IN OIL,DRND SOL | 29.13 | 8.21 | 0 | 198 | 0.727 | 0.629 |
| ATÚN ENLATADO EN AGUA | FISH,TUNA,LT,CND IN H2O,DRND SOL | 19.44 | 0.96 | 0 | 86 | 1.137 | 0.983 |
| BACALAO SECO Y SALADO | COD,ATLANTIC,DRIED&SALTED | 62.82 | 2.37 | 0 | 290 | 0.639 | 0.553 |
| BAGRE | CATFISH,CHANNEL,WILD,RAW | 16.38 | 2.82 | 0 | 95 | 0.773 | 0.668 |
| BANDERON | SWORDFISH,RAW | 19.66 | 6.65 | 0 | 144 | 0.911 | 0.787 |
| BONITO | TUNA,FRESH,BLUEFIN,RAW | 23.33 | 4.9 | 0 | 144 | 0.558 | 0.498 |
| CALAMAR | SQUID,MIXED SPECIES,RAW | 15.58 | 1.38 | 3.08 | 92 | - | - |
| CAMARON | CRUSTACEANS,SHRIMP,UNTREATED,RAW | 20.1 | 0.51 | 0 | 85 | - | - |
| CAMARON COCIDO | CRUSTACEANS,SHRIMP,UNTREATED,CKD | 23.98 | 0.28 | 0.2 | 99 | - | - |
| CANGREJO | CRAB,QUEEN,RAW | 18.5 | 1.18 | 0 | 90 | 0.773 | 0.668 |
| CANGREJO ENLATADO | CRAB,BLUE,CANNED | 17.88 | 0.74 | 0 | 83 | 0.694 | 0.6 |
| CARACOLES (CHUROS) | SNAIL,RAW | 16.1 | 1.4 | 2 | 90 | 0.68 | 0.586 |
| CORVINA | CROAKER,ATLANTIC,RAW | 17.78 | 3.17 | 0 | 104 | 0.625 | 0.494 |
| LANGOSTA | LOBSTER,NORTHERN,RAW | 16.52 | 0.75 | 0 | 77 | 0.508 | 0.479 |
| LANGOSTINO | CRAYFISH,MXD SP,FARMED,RAW | 14.85 | 0.97 | 0 | 72 | 0.756 | 0.654 |
| LENGUADO | FLATFISH (FLOUNDER&SOLE SP),RAW | 12.41 | 1.93 | 0 | 70 | 0.339 | 0.302 |
| OSTIONES | OYSTER,PACIFIC,RAW | 9.45 | 2.3 | 4.95 | 81 | 0.721 | 0.624 |
| PAMPANITO | POMPANO,FLORIDA,RAW | 18.48 | 9.47 | 0 | 164 | 0.801 | 0.692 |
| PARGO | SNAPPER,MIXED SPECIES,RAW | 20.51 | 1.34 | 0 | 100 | 2.452 | 2.121 |
| PESCADO (HUEVOS) | ROE,MIXED SPECIES,RAW | 22.32 | 6.42 | 1.5 | 143 | 0.911 | 0.787 |
| PESCADO DORADO | SWORDFISH,RAW | 19.66 | 6.65 | 0 | 144 | 0.534 | 0.477 |
| PESCADO MERO | GROUPER,MIXED SPECIES,RAW | 19.38 | 1.02 | 0 | 92 | 0.71 | 0.62 |
| PESCADO SIERRA | MACKEREL,PACIFIC&JACK,MXD SP,RAW | 20.07 | 7.89 | 0 | 158 | 0.874 | 0.971 |
| PESCADO TOYO | SHARK,MIXED SPECIES,RAW | 20.98 | 4.51 | 0 | 130 | 0.961 | 0.831 |
| PICUDO | TUNA,FRESH,BLUEFIN,RAW | 23.33 | 4.9 | 0 | 144 | 0.793 | 0.625 |
| PULPO | OCTOPUS,COMMON,RAW | 14.91 | 1.04 | 2.2 | 82 | 0.783 | 0.678 |
| RÓBALO | RÓBALO, PESCADO FRESCO | 18.20 | 0.70 | 0.00 | 84 | 0.81 | 0.68 |
| SALMÓN | SALMON,SOCKEYE,RAW | 21.31 | 5.61 | 0 | 142 | 0.819 | 0.708 |
| SARDINAS CON HUESO ENLATADAS EN ACEITE | SARDINE,ATLANTIC,CND IN OIL,DRND SOL W/BONE | 24.62 | 11.45 | 0 | 208 | 0.811 | 0.701 |
| SARDINAS CON HUESO ENLATADAS EN TOMATE | SARDINE,PACIFIC,CND IN TOMATO SAU,DRND SOL W/BONE | 20.86 | 10.45 | 0.54 | 185 | 0.996 | 0.861 |
| TILAPIA | FISH,TILAPIA,RAW | 20.08 | 1.7 | 0 | 96 | 0.946 | 0.81 |
| TRUCHA | TROUT,MIXED SPECIES,RAW | 20.77 | 6.61 | 0 | 148 | 0.708 | 0.658 |
|  | |  |  |  |  |  |  |
| AGUJA DE RES | BEEF,CHUCK FOR STEW,LN & FAT,SEL,RAW | 21.9 | 3.99 | 0.21 | 124 | 0.854 | 0.777 |
| BIFE CHORIZO | BEEF,COMP OF RTL CUTS,LN&FAT,1/8"FAT,SEL,RAW | 18.87 | 15.75 | 0 | 223 | 0.737 | 0.634 |
| BISTEC DE RES | BEEF,COMP OF RTL CUTS,LN&FAT,1/8"FAT,SEL,RAW | 18.87 | 15.75 | 0 | 223 | 0.737 | 0.634 |
| BORREGO (CORAZÓN) | LAMB,VAR MEATS&BY-PRODUCTS,HEART,RAW | 16.47 | 5.68 | 0.21 | 122 | 0.712 | 0.513 |
| BORREGO (PIERNA) | LAMB,DOM,LEG,WHL (SHK&SIRL),LN&FAT,1/4"FAT,CHOIC,RAW | 17.91 | 17.07 | 0 | 230 | 0.729 | 0.602 |
| BORREGO (RIÑÓN) | LAMB,VAR MEATS&BY-PRODUCTS,KIDNEYS,RAW | 15.74 | 2.95 | 0.82 | 97 | 0.729 | 0.554 |
| CABEZA COCIDA DE CARNERO | CABEZA COCIDA DE CARNERO | 14.20 | 5.60 | 0.00 | 111 | - | - |
| CANE DE RES (FALDA) | BEEF,FLANK,STEAK,LN & FAT,0" FAT,SEL,RAW | 21.22 | 6.06 | 0 | 145 | 0.724 | 0.573 |
| CARNE DE CARNERO | LAMB,DOM,LOIN,LN&FAT,1/4"FAT,CHOIC,RAW | 16.32 | 26.63 | 0 | 310 | 0.626 | 0.472 |
| CARNE DE CARNERO (MAGRA CON HUESO) | LAMB,NZ,IMP,FRZ,RIB,LN,RAW | 20.49 | 6.06 | 0 | 142 | 0.654 | 0.563 |
| CARNE DE CARNERO (MAGRA SIN HUESO) | LAMB,AUS,IMP,FRSH,LEG,SIRLOIN HALF,BNLESS,LN,1/8"FAT,RAW | 20.48 | 5.64 | 0 | 138 | 0.63 | 0.54 |
| CARNE DE CERDO (COSTILLA) | PORK,FRSH,SPARERIBS,LN&FAT,RAW | 15.47 | 23.4 | 0 | 277 | 0.8 | 0.728 |
| CARNE DE CERDO (LOMO, ESPALDILLA Y COSTILLA) | PORK,FRSH,COMP (LEG,LOIN,SHLDR,&SPARERIBS),LN&FAT,RAW | 18.95 | 14.95 | 0 | 216 | 0.645 | 0.555 |
| CARNE DE CERDO (PIERNA O LOMO SIN GRASA) | PORK,FRSH,SHLDR,ARM PICNIC,LN,RAW | 20.26 | 3.77 | 0 | 120 | 1.135 | 0.916 |
| CARNE DE CERDO (SEMIGRASOSA) | PORK,FRSH,COMP (LEG,LOIN,SHLDR,&SPARERIBS),LN&FAT,RAW | 18.95 | 14.95 | 0 | 216 | 0.737 | 0.634 |
| CARNE DE RES (ALTA EN GRASA CON HUESO) | BEEF,RIB,WHL (RIBS 6-12),LN&FAT,1/8"FAT,SEL,RAW | 16.75 | 23.95 | 0 | 288 | 0.664 | 0.549 |
| CARNE DE RES (ALTA EN GRASA SIN HUESO) | CARNE DE RES GRASOSA SIN HUESO | 16.10 | 24.70 | 0.00 | 291 | 0.842 | 0.803 |
| CARNE DE RES (COSTILLAS) | BEEF,RIB,LRG END (RIBS 6-9),LN&FAT,1/8"FAT,SEL,RAW | 16.52 | 24.85 | 0 | 295 | 0.834 | 0.688 |
| CARNE DE RES (FILETE) | BEEF,COMP OF RTL CUTS,LN&FAT,1/8"FAT,SEL,RAW | 18.87 | 15.75 | 0 | 223 | 0.832 | 0.688 |
| CARNE DE RES (MAGRA) | BEEF,CHUCK EYE RST,BNLESS,AMERICA'S BF RST,LN,0" FAT,SEL,RAW | 20.52 | 5.71 | 0 | 133 | 0.753 | 0.648 |
| CARNE DE RES (MOLIDA ESPECIAL) | BEEF,GROUND,85% LN MEAT / 15% FAT,RAW | 18.59 | 15 | 0 | 215 | 0.753 | 0.648 |
| CARNE DE RES (MOLIDA REGULAR) | BEEF,GROUND,75% LN MEAT / 25% FAT,RAW | 15.76 | 25 | 0 | 293 | 0.877 | 0.786 |
| CARNE DE RES (SECA Y CURADA) | BEEF,CURED,DRIED | 31.1 | 1.94 | 2.76 | 153 | 1.135 | 0.916 |
| CARNE DE RES (T- BONE) | BEEF,SHRT LOIN,T-BONE STEAK,LN&FAT,1/8"FAT,SEL,RAW | 19.89 | 11.84 | 0 | 192 | 0.547 | 0.454 |
| CARNE DE RES SEMIGRASOSA | BEEF,COMP OF RTL CUTS,LN&FAT,1/8"FAT,SEL,RAW | 18.87 | 15.75 | 0 | 223 | - | - |
| CARNERO (HÍGADO) | LAMB,VAR MEATS&BY-PRODUCTS,LIVER,RAW | 20.38 | 5.02 | 1.78 | 139 | 0.753 | 0.648 |
| CARNERO (PATAS COCIDAS) | PATAS COCIDAS DE CARNERO | 21.00 | 2.10 | 0.00 | 109 | - | - |
| CECINA DE CERDO | PORK,FRSH,COMP OF RTL CUTS (LEG,LOIN,SHLDR),LN,RAW | 21.2 | 4.82 | 0 | 134 | 0.849 | 0.731 |
| CECINA DE RES (SECA Y CURADA) | BEEF,CURED,DRIED | 31.1 | 1.94 | 2.76 | 153 | - | - |
| CERDO (CHULETA) | PORK,FRSH,COMP (LEG,LOIN,SHLDR,&SPARERIBS),LN&FAT,RAW | 18.95 | 14.95 | 0 | 216 | - | - |
| CERDO (ESPALDILLA) | PORK,FRSH,SHLDR,WHL,LN&FAT,RAW | 17.18 | 17.99 | 0 | 236 | 0.65 | 0.589 |
| CERDO (ESTÓMAGO) | PORK,FRSH,BELLY,RAW | 9.34 | 53.01 | 0 | 518 | 0.681 | 0.579 |
| CERDO (HÍGADO) | PORK,FRSH,VAR MEATS&BY-PRODUCTS,LIVER,RAW | 21.39 | 3.65 | 2.47 | 134 | 0.838 | 0.676 |
| CERDO (PATAS) | PORK,FRSH,VAR MEATS&BY-PRODUCTS,FEET,RAW | 23.16 | 12.59 | 0 | 212 | 0.737 | 0.634 |
| CERDO (PULMÓN) | PORK,FRSH,VAR MEATS&BY-PRODUCTS,LUNGS,RAW | 14.08 | 2.72 | 0 | 85 | 0.665 | 0.508 |
| CERDO (RIÑONES) | PORK,FRSH,VAR MEATS&BY-PRODUCTS,KIDNEYS,RAW | 16.46 | 3.25 | 0 | 100 | 0.688 | 0.627 |
| CERDO (VÍSCERAS) | PORK,FRSH,VAR MEATS&BY-PRODUCTS,CHITTERLINGS,RAW | 7.64 | 16.61 | 0 | 182 | 0.824 | 0.653 |
| CHONTACURO (MAYÓN) | GUSANO MAGUEY | 16.70 | 13.60 | 0.00 | 190 | 1.084 | 0.807 |
| CHORIZO | CHORIZO,PORK AND BEEF | 24.1 | 38.27 | 1.86 | 455 | 0.791 | 0.584 |
| CONEJO | GAME MEAT,RABBIT,DOMESTICATED,COMP OF CUTS,RAW | 20.05 | 5.55 | 0 | 136 | 0.791 | 0.584 |
| GANSO | GOOSE,DOMESTICATED,MEAT&SKN,RAW | 15.86 | 33.62 | 0 | 371 | - | - |
| JAMÓN DE CERDO | PORK,CURED,HAM,WHL,LN&FAT,UNHTD | 18.49 | 18.52 | 0.06 | 246 | 0.615 | 0.482 |
| JAMON DE PAVO | LOUIS RICH,TURKEY HAM (10% H2O) | 18.2 | 4.1 | 0.9 | 113 | - | - |
| JAMÓN DE PAVO | LOUIS RICH,TURKEY HAM (10% H2O) | 18.2 | 4.1 | 0.9 | 113 | 0.477 | 0.411 |
| JAMONADA | PORK,CURED,HAM,WHL,LN&FAT,UNHTD | 18.49 | 18.52 | 0.06 | 246 | - | - |
| LIEBRE | LIEBRE | 21.00 | 5.00 | 0.00 | 135 | 0.82 | 0.34 |
| LONGANIZA | SAUSAGE,ITALIAN,PORK,RAW | 14.25 | 31.33 | 0.65 | 346 | 0.598 | 0.53 |
| MENUDO DE RES (ESTOMAGO E INTESTINOS) | BEEF,VAR MEATS&BY-PRODUCTS,TRIPE,RAW | 12.07 | 3.69 | 0 | 85 | 0.585 | 0.482 |
| MORCILLA (TODAS LAS MARCAS Y CASERA) | BLOOD SAUSAGE | 14.6 | 34.5 | 1.29 | 379 | 0.773 | 0.789 |
| MORTADELA | MORTADELLA,BEEF,PORK | 16.37 | 25.39 | 3.05 | 311 | 0.52 | 0.45 |
| MORTADELA BOLOGÑA | BOLOGNA,PORK | 15.3 | 19.87 | 0.73 | 247 | - | - |
| OSOBUCO | BEEF,SHANK CROSSCUTS,LN,1/4"FAT,CHOIC,RAW | 21.75 | 3.85 | 0 | 128 | - | - |
| PALOMA | SQUAB,(PIGEON),MEAT&SKN,RAW | 18.47 | 23.8 | 0 | 294 | 0.459 | 0.395 |
| PATÉ DE HÍGADO | PATE,LIVER,NOT SPECIFIED,CND | 14.2 | 28 | 1.5 | 319 | 0.582 | 0.454 |
| PATO | DUCK,DOMESTICATED,MEAT&SKN,RAW | 11.49 | 39.34 | 0 | 404 | 0.778 | 0.661 |
| PAVO | TURKEY,WHL,MEAT & SKN,RAW | 21.64 | 5.64 | 0 | 141 | 0.851 | 0.719 |
| PEPPERONI | PEPPERONI,PORK,BEEF | 22.68 | 43.98 | 0 | 494 | 0.696 | 0.561 |
| POLLO | CHICKEN,BROILERS OR FRYERS,MEAT & SKN,RAW | 18.6 | 15.06 | 0 | 215 | 0.62 | 0.569 |
| POLLO (ALAS) | CHICKEN,BROILERS OR FRYERS,WING,MEAT&SKN,RAW | 18.33 | 15.97 | 0 | 222 | 0.816 | 0.684 |
| POLLO (CORAZÓN) | CHICKEN,HEART,ALL CLASSES,RAW | 15.55 | 9.33 | 0.71 | 153 | 0.631 | 0.574 |
| POLLO (HÍGADO) | CHICKEN,LIVER,ALL CLASSES,RAW | 16.92 | 4.83 | 0.73 | 119 | 0.721 | 0.597 |
| POLLO (HÍGADO) | CHICKEN,LIVER,ALL CLASSES,RAW | 16.92 | 4.83 | 0.73 | 119 | 0.696 | 0.557 |
| POLLO (MUSLOS) | CHICKEN,BROILERS OR FRYERS,THIGH,MEAT&SKN,RAW | 16.24 | 16.61 | 0.25 | 219 | 0.824 | 0.653 |
| POLLO (PECHUGA CON PIEL) | CHICKEN,BROILERS OR FRYERS,BREAST,MEAT&SKN,RAW | 20.85 | 9.25 | 0 | 172 | 0.658 | 0.365 |
| POLLO (PIERNA CON PIEL) | CHICKEN,BROILERS OR FRYERS,LEG,MEAT&SKN,RAW | 16.37 | 15.95 | 0.17 | 214 | 0.586 | 0.398 |
| POLLO (PULMÓN) | BEEF,VAR MEATS&BY-PRODUCTS,LUNGS,RAW | 16.2 | 2.5 | 0 | 92 | - | - |
| QUESO DE PUERCO | HEADCHEESE,PORK | 13.83 | 10.9 | 0 | 157 | 0.654 | 0.563 |
| RES (CORAZÓN) | BEEF,VAR MEATS&BY-PRODUCTS,HEART,RAW | 17.72 | 3.94 | 0.14 | 112 | 0.821 | 0.771 |
| RES (HÍGADO) | BEEF,VAR MEATS&BY-PRODUCTS,LIVER,RAW | 20.36 | 3.63 | 3.89 | 135 | 0.777 | 0.592 |
| RES (LENGUA) | BEEF,VAR MEATS&BY-PRODUCTS,TONGUE,RAW | 14.9 | 16.09 | 3.68 | 224 | - | - |
| RES (PATA) | PATA DE RES | 13.30 | 2.20 | 0.00 | 76 | 0.481 | 0.552 |
| RES (PESCUEZO) | BEEF,CHUCK,CLOD RST,LN,1/4" FAT,ALL GRDS,RAW | 19.63 | 5.02 | 0 | 129 | 0.714 | 0.617 |
| RES (RIÑONES) | BEEF,VAR MEATS&BY-PRODUCTS,KIDNEYS,RAW | 17.4 | 3.09 | 0.29 | 99 | 0.445 | 0.376 |
| RES (SESOS) | BEEF,VAR MEATS&BY-PRODUCTS,BRAIN,RAW | 10.86 | 10.3 | 1.05 | 143 | 0.592 | 0.507 |
| RES (TRIPAS) | BEEF,VAR MEATS&BY-PRODUCTS,TRIPE,RAW | 12.07 | 3.69 | 0 | 85 | 0.513 | 0.391 |
| RES (UBRES) | UBRE DE RES | 15.40 | 18.70 | 0.00 | 234 | - | - |
| RIB STEAK | BEEF,RIB,WHL (RIBS 6-12),LN&FAT,1/8"FAT,SEL,RAW | 16.75 | 23.95 | 0 | 288 | - | - |
| RIBEYE STEAK | BEEF,RIB EYE STEAK,BNLESS,LIP OFF,LN & FAT,0" FAT,SEL,RAW | 20.12 | 14.14 | 0 | 208 | - | - |
| SALAMI | SALAMI,CKD,BF&PORK | 21.85 | 25.9 | 2.4 | 336 | - | - |
| SALCHICHA | FRANKFURTER MEAT | 10.26 | 25.76 | 4.17 | 290 | 0.776 | 0.668 |
| SALCHICHA DE PAVO | FRANKFURTER,TURKEY | 12.23 | 17.29 | 3.81 | 223 | 0.781 | 0.617 |
| SALCHICHA DE POLLO | FRANKFURTER,CHICK | 15.51 | 16.19 | 2.74 | 223 | 0.524 | 0.5 |
| SALCHICHAS DE POLLO O RES BAJA EN GRASA | FRANKFURTER,MEAT & POULTRY,LO FAT | 15.5 | 2.8 | 8.4 | 121 | - | - |
| SALMÓN AHUMADO | SALMON,CHINOOK,SMOKED | 18.28 | 4.32 | 0 | 117 | - | - |
| TERNERA | VEAL,COMP OF RTL CUTS,LN&FAT,RAW | 19.35 | 6.77 | 0 | 144 | - | - |
| TOCINO | PORK,CURED,BACON,UNPREP | 12.62 | 39.69 | 1.28 | 417 | 1.149 | 0.748 |
| TOFU | TOFU,RAW,REG,PREP W/CA SULFATE | 8.08 | 4.78 | 1.88 | 76 | 0.393 | 0.27 |
|  | |  |  |  |  |  |  |
| AVENA CON LECHE (NESTLE) |  | 2.50 | 1.50 | 13.00 | 75 | - | - |
| AVENA FINESSE ALPINA |  | 2.00 | 0.60 | 7.60 | 44 | - | - |
| CREMA AGRIA | CREMA 40 % GRASA (AGRIA) | 2.10 | 39.90 | 2.80 | 370 | 0.10 | 0.10 |
| CREMA DE LECHE | CREAM,FLUID,HVY WHIPPING | 2.05 | 37 | 2.79 | 345 | 0.175 | 0.17 |
| LECHE 1% GRASA CON VIT A Y D | MILK,LOWFAT,FLUID,1% MILKFAT,W/ ADDED VIT A & VITAMIN D | 3.37 | 0.97 | 4.99 | 42 | 0.163 | 0.159 |
| LECHE 2% GRASA CON VIT A Y D | MILK,RED FAT,FLUID,2% MILKFAT,W/ ADDED VIT A & VITAMIN D | 3.3 | 1.98 | 4.8 | 50 | 0.046 | 0.053 |
| LECHE CON CHOCOLATE | MILK,CHOC,FLUID,COMM,WHL,W/ ADDED VIT A & VITAMIN D | 3.17 | 3.39 | 10.34 | 83 | 0.171 | 0.167 |
| LECHE CONDENSADA | MILK,CND,COND,SWTND | 7.91 | 8.7 | 54.4 | 321 | 0.174 | 0.17 |
| LECHE DE CABRA | MILK,GOAT,FLUID,W/ ADDED VITAMIN D | 3.56 | 4.14 | 4.45 | 69 | 0.155 | 0.179 |
| LECHE DESCREMADA | MILK,NONFAT,FLUID,WO/ ADDED VIT A & VIT D (FAT FREE OR SKIM) | 3.37 | 0.08 | 4.96 | 34 | 0.329 | 0.329 |
| LECHE EN POLVO | MILK,DRY,WHL,W/ ADDED VITAMIN D | 26.32 | 26.71 | 38.42 | 496 | 0.382 | 0.382 |
| LECHE ENTERA | MILK,WHL,3.25% MILKFAT,WO/ ADDED VIT A & VITAMIN D | 3.15 | 3.27 | 4.78 | 61 | 1.271 | 1.271 |
| LECHE EVAPORADA | MILK,CND,EVAP,W/ ADDED VITAMIN D & WO/ ADDED VIT A | 6.81 | 7.56 | 10.04 | 134 | 0.164 | 0.16 |
| LECHE MATERNA | MILK,HUMAN,MATURE,FLUID | 1.03 | 4.38 | 6.89 | 70 | 0.099 | 0.099 |
| QUESO AZUL | CHEESE,BLUE | 21.4 | 28.74 | 2.34 | 353 | 1.105 | 1.145 |
| QUESO BRIE | CHEESE,BRIE | 20.75 | 27.68 | 0.45 | 334 | 1.311 | 1.202 |
| QUESO CAMEMBERT | CHEESE,CAMEMBERT | 19.8 | 24.26 | 0.46 | 300 | 0.668 | 0.66 |
| QUESO CHEDDAR | CHEESE,CHEDDAR | 24.9 | 33.14 | 1.28 | 403 | 0.577 | 0.604 |
| QUESO COTTAGE | CHEESE,COTTAGE,CRMD,LRG OR SML CURD | 11.12 | 4.3 | 3.38 | 98 | 0.291 | 0.303 |
| QUESO COTTAGE BAJO EN GRASA | CHEESE,COTTAGE,LOWFAT,1% MILKFAT | 12.39 | 1.02 | 2.72 | 72 | - | - |
| QUESO CREMA | CHEESE,CREAM | 5.93 | 34.24 | 4.07 | 342 | - | - |
| QUESO FRESCO | CHEESE,FRSH,QUESO FRESCO | 18.09 | 23.82 | 2.98 | 299 | 1.434 | 1.457 |
| QUESO FRESCO LIGHT | CHEESE,PAST PROCESS,AMERICAN,LOFAT | 24.6 | 7 | 3.5 | 180 | 1.431 | 1.454 |
| QUESO GOUDA | CHEESE,GOUDA | 24.94 | 27.44 | 2.22 | 356 | 1.289 | 1.182 |
| QUESO HOLANDÉS | CHEESE,EDAM | 24.99 | 27.8 | 1.43 | 357 | 1.011 | 1.043 |
| QUESO MANCHEGO | CHEESE,MONTEREY | 24.48 | 30.28 | 0.68 | 373 | 1.266 | 1.403 |
| QUESO MOZARELLA | CHEESE,MOZZARELLA,WHL MILK | 22.17 | 22.35 | 2.19 | 300 | 1.922 | 1.995 |
| QUESO MOZARELLA BAJO EN GRASA | CHEESE,MOZZARELLA,PART SKIM MILK | 24.26 | 15.92 | 2.77 | 254 | 0.556 | 0.589 |
| QUESO PARMESANO | CHEESE,PARMESAN,HARD | 35.75 | 25.83 | 3.22 | 392 | 0.562 | 0.596 |
| QUESO RICOTTA | CHEESE,RICOTTA,WHOLE MILK | 11.26 | 12.98 | 3.04 | 174 | 1.662 | 1.693 |
| QUESO RICOTTA BAJO EN GRASA | CHEESE,RICOTTA,PART SKIM MILK | 11.39 | 7.91 | 5.14 | 138 | - | - |
| QUESO ROQUEFORT | CHEESE,ROQUEFORT | 21.54 | 30.64 | 2 | 369 | 1.023 | 1.012 |
| QUESO SUIZO | CHEESE,SWISS | 26.93 | 27.8 | 5.38 | 380 | 1.919 | 1.852 |
| QUESO TIERNO | CHEESE,FRSH,QUESO FRESCO | 18.09 | 23.82 | 2.98 | 299 | 1.929 | 1.851 |
| YOGURT DE FRUTA | YOGURT,FRUIT,LOFAT,9 GRAMS PROT PER 8 OZ | 3.98 | 1.15 | 18.64 | 99 | 0.286 | 0.265 |
| YOGURT NATURAL | YOGURT,PLN,WHL MILK,8 GRAMS PROT PER 8 OZ | 3.47 | 3.25 | 4.66 | 61 | 0.189 | 0.175 |
| YOGURT NATURAL (BAJO EN GRASA) | YOGURT,PLN,LOFAT,12 GRAMS PROT PER 8 OZ | 5.25 | 1.55 | 7.04 | 63 | 0.217 | 0.201 |
| YOGURT NATURAL DESCREMADO | YOGURT,PLN,SKIM MILK,13 GRAMS PROT PER 8 OZ | 5.73 | 0.18 | 7.68 | 56 | 0.313 | 0.289 |
|  | |  |  |  |  |  |  |
| ACEITE DE AGUACATE | OIL,AVOCADO | 0 | 100 | 0 | 884 | - | - |
| ACEITE DE AJONJOLI | OIL,SESAME,SALAD OR COOKING | 0 | 100 | 0 | 884 | 0 | 0 |
| ACEITE DE CANOLA | OIL,CANOLA | 0 | 100 | 0 | 884 | - | - |
| ACEITE DE COCO | OIL,COCNT | 0 | 100 | 0 | 862 | 0 | 0 |
| ACEITE DE GIRASOL | OIL,SUNFLOWER,LINOLEIC (LESS THAN 60%) | 0 | 100 | 0 | 884 | 0 | 0 |
| ACEITE DE MAÍZ | OIL,CORN,INDUSTRIAL & RTL,ALLPURP SALAD OR COOKING | 0 | 100 | 0 | 900 | 0 | 0 |
| ACEITE DE OLIVA | OIL,OLIVE,SALAD OR COOKING | 0 | 100 | 0 | 884 | 0 | 0 |
| ACEITE DE PALMA | OIL,PALM | 0 | 100 | 0 | 884 | 0 | 0 |
| ACEITE DE SOYA | OIL,SOYBN,SALAD OR COOKING | 0 | 100 | 0 | 884 | 0 | 0 |
| MANTECA DE CERDO | LARD | 0 | 100 | 0 | 902 | 0 | 0 |
| MANTEQUILLA CON SAL | BUTTER,WITH SALT | 0.85 | 81.11 | 0.06 | 717 | 0.041 | 0.041 |
| MANTEQUILLA BAJA EN GRASA | BUTTER,LT,STK,W/SALT | 3.3 | 55.1 | 0 | 509 | - | - |
| MANTEQUILLA SIN SAL | BUTTER,WITHOUT SALT | 0.85 | 81.11 | 0.06 | 717 | 0.041 | 0.041 |
| MARGARINA BAJA EN GRASA | MARGARINE SPRD,APPROX 48% FAT,TUB | 0.2 | 47.53 | 0.86 | 424 | - | - |
| MARGARINA | MARGARINE,REG,80% FAT,COMP,TUB,W/ SALT | 0.22 | 80.17 | 0.75 | 713 | - | - |
|  | |  |  |  |  |  |  |
| ACHIOTE | ACHIOTE | 11.3 | 5.3 | 75.9 | 388 | - | - |
| CANELA | CINNAMON,GROUND | 3.99 | 1.24 | 80.59 | 247 | 0.449 | 0.297 |
| CILANTRO | CORIANDER SEED | 12.37 | 17.77 | 54.99 | 298 | 0.996 | 0.758 |
| CLAVO DE OLOR | CLOVES,GROUND | 5.97 | 13 | 65.53 | 274 | 2.08 | 1.844 |
| COMINO | CUMIN SEED | 17.81 | 22.27 | 44.24 | 375 | - | - |
| CURRY EN POLVO | CURRY POWDER | 14.29 | 14.01 | 55.83 | 325 | 0.89 | 0.7 |
| ENELDO | DILL WEED,DRIED | 19.96 | 4.36 | 55.82 | 253 | 0.446 | 0.483 |
| GENJIBRE | GINGER,GROUND | 8.98 | 4.24 | 71.62 | 335 | 0.92 | 0.69 |
| HINOJO SEMILLA | FENNEL SEED | 15.8 | 14.87 | 52.29 | 345 | - | - |
| NUEZ MOSCADA | NUTMEG,GROUND | 5.84 | 36.31 | 49.29 | 525 | 0.513 | 0.241 |
| ORÉGANO | SPICES,OREGANO,DRIED | 9 | 4.28 | 68.92 | 265 | - | - |
| PAPRIKA | PAPRIKA | 14.14 | 12.89 | 53.99 | 282 | 0.4 | 0.37 |
| PIMIENTA | PEPPER,BLACK | 10.39 | 3.26 | 63.95 | 251 | - | - |
| SAL | SALT,TABLE | 0 | 0 | 0 | 0 | 0 | 0 |
| SEMILLA DE MOSTAZA | SPICES,MUSTARD SD,GROUND | 26.08 | 36.24 | 28.09 | 508 | 0.146 | 0.136 |
|  | |  |  |  |  |  |  |
| ADEREZO PARA ENSALADA RANCH REGULAR | SALAD DRSNG,RANCH DRSNG,COMM,REG | 1.03 | 51.39 | 6.69 | 484 | - | - |
| ADEREZO PARA ENSALADA THOUSAND ISLAND | SALAD DRSNG,1000 ISLAND,COMM,REG | 1.09 | 35.06 | 14.64 | 370 | 1.75 | 1.13 |
| ADEREZO PARA ENSALADA VINAGRETA BALSÁMICA | VINEGAR,BALSAMIC | 0.49 | 0 | 17.03 | 88 | - | - |
| ADEREZO PARA ENSALADA VINAGRETA ITALIANA REGULAR | SALAD DRSNG,ITALIAN DRSNG,COMM,REG | 0.41 | 21.12 | 12.12 | 240 | 0 | 0 |
| BOCADITOS DE CHICHARRON | PORK SKINS,PLAIN | 61.3 | 31.3 | 0 | 544 | 0.25 | 0.158 |
| BOCADITOS DE MAÍZ TIPO DORITOS, RISKOS, ETC | CORN-BASED,EXTRUDED,CHIPS,PLN | 6.03 | 28.41 | 63.01 | 518 | 0.262 | 0.165 |
| BOCADITOS DE MAÍZ SABOR A QUESO | CORN-BASED,EXTRUDED,PUFFS OR TWISTS,CHEESE-FLAVOR | 5.87 | 36.56 | 53 | 563 | - | - |
| CAFÉ INSTANTÁNEO CON AZÚCAR Y SABOR CAPUCCINO EN POLVO | COFFEE,INST,W/SUGAR,CAPPUCCINO-FLAVOR PDR | 3.1 | 5.56 | 85.94 | 406 | - | - |
| CAFÉ INSTANTÁNEO DESCAFEINADO EN POLVO | COFFEE,INST,DECAFFEINATED,PDR | 11.6 | 0.2 | 76 | 351 | - | - |
| CAFÉ INSTANTANEO REGULAR EN POLVO | COFFEE,INST,REG,PDR | 12.2 | 0.5 | 75.4 | 353 | 0 | 0 |
| CHIFLES DE SAL TIPO TORTOLINES, BANCHIS, ETC | SNACKS,PLANTAIN CHIPS,SALTED | 2.28 | 29.59 | 63.84 | 531 | - | - |
| COMPOTA DE VERDURAS TIPO GERBER | BABYFOOD,DINNER,VEG&DUMPLINGS&BF,STR | 2 | 0.9 | 7.7 | 48 | 0.011 | 0.005 |
| COMPOTA FRUTAS MIXTAS TIPO GERBER | BABYFOOD,FRUIT,PEARS&PNAPPL,STR | 0.3 | 0.1 | 10.9 | 41 | - | - |
| CREMA PARA CAFÉ COFFE MATTE Y OTRAS MARCAS |  | 0.00 | 25.00 | 50.00 | 500 | 0.057 | 0.046 |
| ENSURE LIQUIDO | ENSURE PLUS,LIQ NUTR | 5.16 | 4.52 | 19.88 | 141 | 0.167 | 0.139 |
| EXTRACTO DE VAINILLA | VANILLA EXTRACT | 0.06 | 0.06 | 12.65 | 288 | 0.13 | 0.066 |
| HABAS FRITAS SALADAS |  | 28.00 | 20.00 | 24.00 | 320 | - | - |
| LEVADURA | LEAVENING AGENTS,YEAST,BAKER'S,ACTIVE DRY | 40.44 | 7.61 | 41.22 | 325 | 0.016 | 0.011 |
| MACARRONES CON QUESO | MACARONI & CHS,BOX MIX W/ CHS SAU,UNPREP | 12.73 | 10.67 | 46.66 | 334 | 0.353 | 0.244 |
| MAYONESA | SALAD DRSNG,MAYO,REG | 0.96 | 74.85 | 0.57 | 680 | 0.026 | 0.011 |
| MAYONESA LIGHT KRAFT | SALAD DRSNG,KRAFT MAYO LT MAYO | 0.6 | 32.9 | 8.5 | 334 | - | - |
| MIX KIWA |  | 2.86 | 28.57 | 62.86 | 514 | - | - |
| MOSTAZA | MUSTARD,PREPARED,YELLOW | 4.37 | 4.01 | 5.33 | 67 | - | - |
| PAPAS FRITAS EN FUNDA | POTATO CHIPS,PLAIN,SALTED | 6.56 | 36.4 | 50.81 | 542 | - | - |
| PASTA DE TOMATE | TOMATO PRODUCTS,CND,PASTE,WO/ SALT ADDED | 4.32 | 0.47 | 18.91 | 82 | - | - |
| PEDIASURE LIQUIDO | CHILD FORMULA,ABBOTT NUTR,PEDIASURE,RTF (FORMERLY ROSS) | 2.86 | 4.77 | 11.15 | 99 | - | - |
| PIZZA CONGELADA | PIZZA,MEAT & VEG TOPPING,REG CRUST,FRZ,CKD | 11.28 | 14.43 | 25.14 | 276 | 0.322 | 0.266 |
| POLVO DE HORNEAR | LEAVENING AGENTS,BAKING PDR,DOUBLE-ACTING,NA AL SULFATE | 0 | 0 | 27.7 | 53 | - | - |
| PRETZELS | PRETZELS,HARD,PLN,SALTED | 10.34 | 2.63 | 79.76 | 380 | - | - |
| SALSA BBQ | SAUCE,BARBECUE | 0.82 | 0.63 | 40.77 | 172 | 1.94 | 1.205 |
| SALSA DE AJÍ | SAUCE,RTS,PEPPER OR HOT | 0.51 | 0.37 | 1.75 | 11 | 0.52 | 0.324 |
| SALSA DE SOYA | SOY SAU MADE FROM SOY&WHEAT (SHOYU) | 8.14 | 0.57 | 4.93 | 53 | 0.31 | 0.259 |
| SALSA DE TOMATE | CATSUP | 1.25 | 0.18 | 26.23 | 112 | 0.366 | 0.288 |
| SALSA INGLESA | SAUCE,WORCESTERSHIRE | 0 | 0 | 19.46 | 78 | - | - |
| SNACK SHULLO |  | 22.86 | 45.71 | 22.86 | 571 | - | - |
| SOPA DE SOBRE EN POLVO | SOUP,CRM OF VEG,DRY,PDR | 8 | 24.1 | 52.1 | 446 | - | - |
| VINAGRE | VINEGAR,CIDER | 0 | 0 | 0.93 | 21 | - | - |
| YUCAS FRITAS EN FUNDA | SNACKS,YUCCA (CASSAVA) CHIPS,SALTED | 1.34 | 25.91 | 69.23 | 515 | - | - |
|  | |  |  |  |  |  |  |
| AZÚCAR BLANCA | SUGARS,GRANULATED | 0 | 0 | 99.98 | 387 | - | - |
| AZÚCAR MORENA | SUGARS,BROWN | 0.12 | 0 | 98.09 | 380 | - | - |
| CARAMELO/ CHUPETE/ GOMITA | CANDIES,CARAMELS | 4.6 | 8.1 | 77 | 382 | - | - |
| CHOCOLATE EN BARRA | CANDIES,MILK CHOC | 7.65 | 29.66 | 59.4 | 535 | - | - |
| COCOA EN POLVO | COCOA MIX,PDR | 6.67 | 4 | 83.73 | 398 | - | - |
| DULCE DE LECHE | CAJETA | 0.10 | 2.80 | 94.90 | 392 | - | - |
| FLAN | FLAN,CARAMEL CUSTARD,DRY MIX,PREP W/ WHL MILK | 2.95 | 3 | 18.68 | 113 | - | - |
| FRUCTOSA LÍQUIDA (EDULCORANTE) | SWEETENERS,TABLETOP,FRUCTOSE,LIQ | 0 | 0 | 76.1 | 279 | 0.011 | 0.008 |
| GELATINA EN POLVO | GELATIN DSSRT,DRY MIX | 7.8 | 0 | 90.5 | 381 | - | - |
| GELATINA PREPARADA | GELATIN DSSRT,DRY MIX,PREP W/ H2O | 1.22 | 0 | 14.19 | 62 | - | - |
| HELADO DE VAINILLA | ICE CREAMS,REG,LO CARBOHYDRATE,VANILLA | 3.17 | 12.7 | 22.23 | 216 | 0.266 | 0.165 |
| JARABE DE MAPLE | SYRUPS,MAPLE | 0.04 | 0.06 | 67.04 | 260 | 0.118 | 0.072 |
| MELAZA | MOLASSES | 0 | 0.1 | 74.73 | 290 | 0.205 | 0.205 |
| MERMELADA | JELLIES | 0.15 | 0.02 | 69.95 | 266 | - | - |
| MERMELADA BAJA EN AZÚCAR | JELLIES,RED SUGAR,HOME PRESERVED | 0.3 | 0.03 | 46.1 | 179 | - | - |
| MIEL DE ABEJA | HONEY | 0.3 | 0 | 82.4 | 304 | - | - |
| MIEL DE CAÑA | SYRUP,CANE | 0 | 0 | 73.14 | 269 | 0.177 | 0.031 |
| MIEL DE MAÍZ | SYRUPS,CORN,DK | 0 | 0 | 77.59 | 286 | 0.028 | 0.005 |
| PANELA | PILONCILLO | 0.00 | 0.00 | 97.30 | 376 | 0.157 | 0.155 |
| PIE DE MANZANA PROMEDIO | PIE,APPL,PREP FROM RECIPE | 2.4 | 12.5 | 37.1 | 265 | 0.143 | 0.143 |
| ROLLOS DE CANELA TIPO CINNABON | SWEET ROLLS,CINN,REFR DOUGH W/FRSTNG,BKD | 5.4 | 13.2 | 56.1 | 362 | - | - |
| SUCRALOSA (EDULCORANTE) | SWEETENERS,TABLETOP,SUCRALOSE,SPLENDA PACKETS | 0 | 0 | 91.17 | 336 | - | - |
| SUSPIROS |  | 12.50 | 37.50 | 50.00 | 625 | - | - |
|  | |  |  |  |  |  |  |
| AGUA AROMÁTICA SIN AZÚCAR | TEA,HERB,OTHER THAN CHAMOMILE,BREWED | 0 | 0 | 0.2 | 1 | - | - |
| AGUA EMBOTELLADA | WATER,BTLD,POLAND SPRING | 0 | 0 | 0 | 0 | - | - |
| AVENA CON FRUTA (NESTLE) |  | 0.50 | 0.50 | 13.00 | 57 | - | - |
| BEBIDA DE MALTA | MALT BEV,INCL NON-ALCOHOLIC BEER | 0.21 | 0.12 | 8.05 | 37 | - | - |
| BEBIDA ENERGIZANTE RED BULL | ENGY DRK,RED BULL,W/ ADD CAFFEINE,NIACIN,PANTO,VIT B6 & B12 | 0.25 | 0.08 | 10.94 | 45 | - | - |
| BEBIDA ENERGIZANTE RED BULL SIN AZUCAR | ENG DRK,RED BULL,SGR FR,W/ADD CAFF,NIACIN,PANTO,VIT B6 & B12 | 0.25 | 0.08 | 0.7 | 5 | 0 | 0 |
| CAFÉ PASADO/ TOSTADO Y MOLIDO | COFFEE,BREWED,ESPRESSO,REST-PREP | 0.12 | 0.18 | 1.67 | 9 | 0 | 0 |
| CERVEZA LIGHT PROMEDIO | ALCOHOLIC BEV,BEER,LT | 0.24 | 0 | 1.64 | 29 | - | - |
| CERVEZA PROMEDIO | ALCOHOLIC BEV,BEER,REG,ALL | 0.46 | 0 | 3.55 | 43 | - | - |
| GASEOSA NEGRA (TODAS LAS MARCAS) | CARBONATED BEV,COLA,CONTAINS CAFFEINE | 0.07 | 0.02 | 9.56 | 37 | 0 | 0 |
| GASEOSA NEGRA CON ASPARTAME (TODAS LAS MARCAS) | CARB BEV,LO CAL,COLA OR PEP,W/ ASPRT, CAF | 0.11 | 0.03 | 0.29 | 2 | - | - |
| GASEOSA SABOR FRESA O MANZANA | CARBONATED BEV,ORANGE | 0 | 0 | 12.3 | 48 | - | - |
| GASEOSA SABOR LIMÓN | CARBONATED BEV,LEMON-LIME SODA,CONTAINS CAFFEINE | 0.09 | 0 | 10.42 | 41 | - | - |
| GASEOSA SABOR LIMÓN CON ASPARTAME | CARB BEV,LO CAL,OTHR THN COLA OR PEPPER,W/ ASPRT,CONTNS CAFF | 0.1 | 0 | 0 | 0 | - | - |
| GASEOSA SABOR NARANJA | CARBONATED BEV,ORANGE | 0 | 0 | 12.3 | 48 | - | - |
| GASEOSA SABOR UVA | CARBONATED BEV,GRAPE SODA | 0 | 0 | 11.2 | 43 | - | - |
| GATORADE | SPORTS DRK,PEPSICO QUAKER GATORADE,ORIGINAL,FRUIT-FLAVD,RTD | 0 | 0 | 6.43 | 26 | - | - |
| GIN | ALCOHOLIC BEV,DISTILLED,GIN,90 PROOF | 0 | 0 | 0 | 263 | - | - |
| JUGO EN POLVO SABOR FRESA (TODAS LAS MARCAS) | STRAWBERRY-FLAVOR BEV MIX,PDR | 0.1 | 0.2 | 99.1 | 389 | - | - |
| JUGO EN POLVO SIN CALORIAS | FRUIT-FLAVORED DRK,DRY PDR MIX,LO CAL,W/ ASPRT | 0.45 | 0.04 | 87.38 | 218 | - | - |
| JUGO HUESITO + CALCIO |  | 0.00 | 0.00 | 11.11 | 44 | - | - |
| JUGO LIGHT TETRAPAK NATURA (TODOS LOS SABORES) |  | 0.45 | 0.00 | 3.22 | 17 | 0 | 0 |
| JUGOS TETRAPAK (NATURA TODOS LOS SABORES) |  | 0.00 | 0.00 | 10.95 | 45 | - | - |
| NECTAR DE FRUTAS TETRAPAK (NATURA) |  | 0.45 | 0.00 | 12.50 | 53 | - | - |
| PENCO (JUGO CHAGUARMISHQUI) | PULQUE | 0.30 | 0.00 | 6.10 | 43 | 0.01 | 0.01 |
| PIÑA COLADA | ALCOHOLIC BEV,PINA COLADA,CND | 0.6 | 7.6 | 27.6 | 237 | 0 | 0 |
| POWERADE | SPORTS DRK,COCA-COLA,POWERADE,LEMON-LIME FLAV,RTD | 0 | 0.05 | 7.84 | 32 | 0 | 0 |
| RON | ALCOHOLIC BEV,DISTILLED,RUM,80 PROOF | 0 | 0 | 0 | 231 | - | - |
| TE HELADO (NESTEA) | TEA,RTD,NESTLE,COOL NESTEA ICE TEA LEMON FLAVOR | 0 | 0 | 9.09 | 36 | - | - |
| TE HELADO EN POLVO | TEA,INST,SWTND W/SUGAR,LEMN-FLAV,WO/ VIT C,PDR,DECAFFEINATED | 0.12 | 0.73 | 98.55 | 401 | 0 | 0 |
| TEQUILA SUNRISE | ALCOHOLIC BEV,TEQUILA SUNRISE,CND | 0.3 | 0.1 | 11.3 | 110 | 0 | 0 |
| VINO BLANCO | ALCOHOLIC BEV,WINE,TABLE,WHITE | 0.07 | 0 | 2.6 | 82 | 0 | 0 |
| VINO ROJO | ALCOHOLIC BEV,WINE,TABLE,RED | 0.07 | 0 | 2.61 | 85 | - | - |
| VODKA | ALCOHOLIC BEV,DISTILLED,VODKA,80 PROOF | 0 | 0 | 0 | 231 | - | - |
| WHISKEY | ALCOHOLIC BEV,DISTILLED,WHISKEY,86 PROOF | 0 | 0 | 0.1 | 250 | - | - |
|  | |  |  |  |  |  |  |
| 1/4 POLLO ASADO CON PAPAS FRITAS (PROMEDIO) | 1/4 POLLO ASADO CON PAPAS FRITAS (PROMEDIO) | 5.20 | 7.83 | 19.11 | 169.49 | 0.2027062 | 0.153901 |
| ALIÑO (PROMEDIO) | ALIÑO (PROMEDIO) | 3.23 | 0.31 | 17.94 | 80.37 | 0.0983872 | 0.0438076 |
| AREPAS DE ZAPALLO (PROMEDIO) | AREPAS DE ZAPALLO (PROMEDIO) | 7.50 | 10.32 | 31.98 | 245.98 | 0.1644537 | 0.1240629 |
| ARROPE DE MORA (PROMEDIO) | ARROPE DE MORA (PROMEDIO) | 0.82 | 0.29 | 30.78 | 122.57 | 0 | 0 |
| ARROZ CON HUEVO FRITO (PROMEDIO) | ARROZ CON HUEVO FRITO (PROMEDIO) | 3.58 | 2.91 | 26.71 | 151.05 | 0.1912906 | 0.1274472 |
| ARROZ CON LECHE (PROMEDIO) | ARROZ CON LECHE (PROMEDIO) | 2.13 | 5.03 | 21.95 | 140.94 | 0.1104855 | 0.0912863 |
| ARROZ RELLENO CON PESCADO (PROMEDIO) | ARROZ RELLENO CON PESCADO (PROMEDIO) | 10.40 | 2.01 | 17.24 | 132.70 | 0.4247362 | 0.3447012 |
| ARROZ RELLENO CON POLLO (PROMEDIO) | ARROZ RELLENO CON POLLO (PROMEDIO) | 7.60 | 8.79 | 15.86 | 174.95 | 0.3193597 | 0.2400089 |
| BALA DE VERDE (PROMEDIO) | BALA DE VERDE (PROMEDIO) | 0.81 | 20.97 | 19.07 | 258.01 | 0.02618 | 0.01904 |
| BISTEC DE HÍGADO (PROMEDIO) | BISTEC DE HÍGADO (PROMEDIO) | 19.56 | 11.00 | 5.06 | 200.17 | 1.035185 | 0.7698481 |
| BOLLO DE CARNE (PROMEDIO) | BOLLO DE CARNE (PROMEDIO) | 10.60 | 7.34 | 13.80 | 154.44 | 0.4163651 | 0.3449199 |
| BOLLO DE CUERO DE CERDO (PROMEDIO) | BOLLO DE CUERO DE CERDO (PROMEDIO) | 6.13 | 11.55 | 10.89 | 165.89 | 0.1061249 | 0.0705544 |
| BOLLO DE MANÍ (PROMEDIO) | BOLLO DE MANÍ (PROMEDIO) | 4.80 | 7.55 | 18.97 | 150.27 | 0.1972901 | 0.1313947 |
| BOLLO DE PESCADO (PROMEDIO) | BOLLO DE PESCADO (PROMEDIO) | 11.29 | 7.68 | 12.84 | 159.55 | 0.4445887 | 0.3585977 |
| BOLÓN DE CHICHARRÓN Y QUESO (PROMEDIO) | BOLÓN DE CHICHARRÓN Y QUESO (PROMEDIO) | 2.26 | 12.41 | 7.22 | 146.31 | 0.0096111 | 0.0069899 |
| BOLÓN DE VERDE CON CARNE DE CERDO (PROMEDIO) | BOLÓN DE VERDE CON CARNE DE CERDO (PROMEDIO) | 7.40 | 5.49 | 17.10 | 141.76 | 0.2893634 | 0.2458081 |
| CALDO DE 31 (PROMEDIO) | CALDO DE 31 (PROMEDIO) | 7.44 | 3.82 | 0.55 | 67.97 | 0.0021849 | 0.0014117 |
| CALDO DE BAGRE (PROMEDIO) | CALDO DE BAGRE (PROMEDIO) | 2.60 | 4.73 | 5.33 | 72.03 | 0.1034523 | 0.0832837 |
| CALDO DE GALLINA CON PAPA (PROMEDIO) | CALDO DE GALLINA CON PAPA (PROMEDIO) | 6.27 | 6.39 | 4.49 | 101.29 | 0.2292212 | 0.1879645 |
| CALDO DE GALLINA CON YUCA (PROMEDIO) | CALDO DE GALLINA CON YUCA (PROMEDIO) | 5.68 | 5.60 | 7.32 | 103.16 | 0.2143177 | 0.1743074 |
| CALDO DE HABAS VERDES CON CHANCHO (PROMEDIO) | CALDO DE HABAS VERDES CON CHANCHO (PROMEDIO) | 6.63 | 6.21 | 3.10 | 95.43 | 0.2630156 | 0.2256131 |
| CALDO DE MANGUERA (PROMEDIO) | CALDO DE MANGUERA (PROMEDIO) | 8.48 | 3.03 | 8.29 | 92.46 | 0.1918225 | 0.1621822 |
| CALDO DE MORCILLA (PROMEDIO) | CALDO DE MORCILLA (PROMEDIO) | 8.44 | 3.37 | 13.79 | 120.97 | 0.2439355 | 0.203946 |
| CALDO DE PATA (PROMEDIO) | CALDO DE PATA (PROMEDIO) | 4.59 | 3.18 | 13.55 | 98.63 | 0.2042446 | 0.1736238 |
| CARNES ASADAS DE SAN JOAQUÍN (PROMEDIO) | CARNES ASADAS DE SAN JOAQUÍN (PROMEDIO) | 7.51 | 5.69 | 13.18 | 133.25 | 0.2690171 | 0.2425871 |
| CARNES COLORADAS (PROMEDIO) | CARNES COLORADAS (PROMEDIO) | 7.41 | 6.98 | 14.78 | 149.20 | 0.2259869 | 0.1951598 |
| CAZUELA DE PESCADO (PROMEDIO) | CAZUELA DE PESCADO (PROMEDIO) | 7.35 | 6.73 | 6.46 | 112.38 | 0.2989817 | 0.2429754 |
| CEBICHE DE CAMARÓN (PROMEDIO) | CEBICHE DE CAMARÓN (PROMEDIO) | 6.33 | 3.92 | 22.66 | 146.26 | 0.1048858 | 0.0835177 |
| CEBICHE DE CHOCHOS (PROMEDIO) | CEBICHE DE CHOCHOS (PROMEDIO) | 2.53 | 3.23 | 13.23 | 88.63 | 0.0335826 | 0.0259146 |
| CEBICHE DE CONCHA (PROMEDIO) | CEBICHE DE CONCHA (PROMEDIO) | 4.11 | 4.71 | 31.98 | 180.77 | 0.169634 | 0.1448022 |
| CEBICHE DE PESCADO (PROMEDIO) | CEBICHE DE PESCADO (PROMEDIO) | 7.59 | 4.65 | 21.64 | 155.38 | 0.2966225 | 0.2499059 |
| CHAMPÚS (PROMEDIO) | CHAMPÚS (PROMEDIO) | 1.28 | 0.67 | 29.22 | 123.26 | 0.0574718 | 0.0473905 |
| CHAULAFÁN (PROMEDIO) | CHAULAFÁN (PROMEDIO) | 8.85 | 6.36 | 16.51 | 161.40 | 0.3549962 | 0.2846115 |
| CHICHA DE CHONTA (PROMEDIO) | CHICHA DE CHONTA (PROMEDIO) | 0.71 | 0.61 | 23.89 | 98.88 | 0 | 0 |
| CHICHA DE MÁCHICA (PROMEDIO) | CHICHA DE MÁCHICA (PROMEDIO) | 1.77 | 0.18 | 32.86 | 128.52 | 0.0016704 | 0.0012611 |
| CHICHA DE YUCA (PROMEDIO) | CHICHA DE YUCA (PROMEDIO) | 0.56 | 0.09 | 22.93 | 93.43 | 0.0150105 | 0.0080107 |
| CHIFLES DE DULCE (PROMEDIO) | CHIFLES DE DULCE (PROMEDIO) | 0.96 | 19.50 | 21.59 | 260.52 | 0 | 0 |
| CHIFLES DE SAL (PROMEDIO) | CHIFLES DE SAL (PROMEDIO) | 0.83 | 16.91 | 20.31 | 225.11 | 0.0280255 | 0.0203822 |
| CHOCLO CON QUESO (PROMEDIO) | CHOCLO CON QUESO (PROMEDIO) | 10.42 | 12.15 | 14.45 | 200.00 | 0.0803419 | 0.0653102 |
| CHUCULA (PROMEDIO) | CHUCULA (PROMEDIO) | 4.01 | 4.46 | 15.38 | 116.69 | 0.1067911 | 0.1041617 |
| CHURRASCO (PROMEDIO) | CHURRASCO (PROMEDIO) | 8.67 | 7.24 | 12.33 | 151.81 | 0.3478062 | 0.2832444 |
| COCADA (PROMEDIO) | COCADA (PROMEDIO) | 4.83 | 15.12 | 12.12 | 199.20 | 0.2278553 | 0.2130906 |
| COLADA DE ARVEJA (PROMEDIO) | COLADA DE ARVEJA (PROMEDIO) | 5.14 | 2.60 | 18.56 | 115.28 | 0.0134442 | 0.0111303 |
| COLADA DE SAMBO (PROMEDIO) | COLADA DE SAMBO (PROMEDIO) | 1.22 | 0.94 | 17.12 | 78.72 | 0.0541631 | 0.0452044 |
| COLADA DE UVILLA (PROMEDIO) | COLADA DE UVILLA (PROMEDIO) | 2.60 | 0.42 | 27.60 | 123.71 | 0.1159716 | 0.0695848 |
| COLADA DE YAGUANA (PROMEDIO) | COLADA DE YAGUANA (PROMEDIO) | 0.26 | 0.09 | 15.11 | 59.28 | 0.0069738 | 0.0044729 |
| COLADA MORADA (PROMEDIO) | COLADA MORADA (PROMEDIO) | 1.00 | 0.50 | 18.52 | 79.02 | 0.0369844 | 0.0311218 |
| CONEJO ASADO (PROMEDIO) | CONEJO ASADO (PROMEDIO) | 12.25 | 7.44 | 7.71 | 145.08 | 0.5011232 | 0.4202098 |
| CORVICHE (PROMEDIO) | CORVICHE (PROMEDIO) | 11.52 | 9.36 | 10.05 | 165.12 | 0.4685165 | 0.3807279 |
| CUCHICHAQUI (PROMEDIO) | CUCHICHAQUI (PROMEDIO) | 7.80 | 13.34 | 32.25 | 276.98 | 0.1796641 | 0.1557941 |
| CUY ASADO (PROMEDIO) | CUY ASADO (PROMEDIO) | 19.20 | 5.38 | 0.66 | 132.89 | 0.7836793 | 0.6788419 |
| DULCE DE GUINEO (PROMEDIO) | DULCE DE GUINEO (PROMEDIO) | 0.75 | 0.22 | 22.70 | 88.49 | 0.0206887 | 0.0058292 |
| EMBORRAJADOS (PROMEDIO) | EMBORRAJADOS (PROMEDIO) | 6.94 | 12.86 | 27.20 | 252.93 | 0.2134014 | 0.1485564 |
| EMPANADA DE CAMARÓN (PROMEDIO) | EMPANADA DE CAMARÓN (PROMEDIO) | 8.43 | 17.65 | 39.73 | 350.02 | 0.2761351 | 0.1708634 |
| EMPANADA DE CARNE (PROMEDIO) | EMPANADA DE CARNE (PROMEDIO) | 10.35 | 20.21 | 34.56 | 361.99 | 0.4573221 | 0.3359191 |
| EMPANADA DE POLLO (PROMEDIO) | EMPANADA DE POLLO (PROMEDIO) | 8.73 | 20.30 | 31.86 | 343.67 | 0.3873323 | 0.2743073 |
| EMPANADAS DE MOROCHO (PROMEDIO) | EMPANADAS DE MOROCHO (PROMEDIO) | 9.66 | 13.54 | 24.08 | 255.19 | 0.4126169 | 0.3040529 |
| EMPANADAS DE VERDE (PROMEDIO) | EMPANADAS DE VERDE (PROMEDIO) | 4.82 | 15.64 | 16.76 | 219.46 | 0.0238532 | 0.0179613 |
| ENCEBOLLADO DE PESCADO (PROMEDIO) | ENCEBOLLADO DE PESCADO (PROMEDIO) | 4.64 | 4.34 | 8.73 | 91.49 | 0.1733144 | 0.143604 |
| ENCOCADO DE PESCADO (PROMEDIO) | ENCOCADO DE PESCADO (PROMEDIO) | 7.97 | 6.66 | 13.48 | 143.82 | 0.3198982 | 0.2623445 |
| ENSALADA DE FRUTAS (PROMEDIO) | ENSALADA DE FRUTAS (PROMEDIO) | 0.73 | 0.23 | 11.43 | 46.95 | 0.0167957 | 0.0089256 |
| ENSALADA DE MELLOCO (PROMEDIO) | ENSALADA DE MELLOCO (PROMEDIO) | 0.96 | 0.14 | 10.13 | 43.20 | 0.0112242 | 0.0072179 |
| ENSUMACADO DE MARISCOS (PROMEDIO) | ENSUMACADO DE MARISCOS (PROMEDIO) | 12.04 | 7.30 | 3.32 | 125.79 | 0.3917124 | 0.3323571 |
| ESTOFADO DE PESCADO (PROMEDIO) | ESTOFADO DE PESCADO (PROMEDIO) | 10.30 | 8.24 | 7.86 | 145.25 | 0.3990896 | 0.3407849 |
| FRITADA (PROMEDIO) | FRITADA (PROMEDIO) | 2.67 | 2.13 | 12.94 | 80.31 | 0.1035967 | 0.0874033 |
| GUATITA (PROMEDIO) | GUATITA (PROMEDIO) | 6.46 | 5.96 | 12.93 | 129.10 | 0.1243588 | 0.0878035 |
| HELADO DE PAILA DE MORA (PROMEDIO) | HELADO DE PAILA DE MORA (PROMEDIO) | 2.79 | 0.31 | 30.09 | 128.80 | 0.1257308 | 0.0837594 |
| HIGOS CON QUESO (PROMEDIO) | HIGOS CON QUESO (PROMEDIO) | 3.93 | 5.04 | 61.96 | 299.46 | 0.0036031 | 0.0064055 |
| HORNADO (PROMEDIO) | HORNADO (PROMEDIO) | 2.80 | 2.70 | 7.43 | 64.16 | 0.0700936 | 0.0579297 |
| HUMITA DE DULCE (PROMEDIO) | HUMITA DE DULCE (PROMEDIO) | 5.95 | 20.64 | 12.87 | 257.61 | 0.1553677 | 0.1294914 |
| HUMITA DE SAL (PROMEDIO) | HUMITA DE SAL (PROMEDIO) | 6.57 | 21.90 | 5.20 | 241.80 | 0.1701759 | 0.1421431 |
| LIBRILLO (PROMEDIO) | LIBRILLO (PROMEDIO) | 6.54 | 5.84 | 12.88 | 128.16 | 0.1274035 | 0.0903514 |
| LLAPINGACHOS CON CHORIZO (PROMEDIO) | LLAPINGACHOS CON CHORIZO (PROMEDIO) | 10.11 | 16.93 | 8.32 | 226.02 | 0.354468 | 0.2353642 |
| LOCRO DE PAPA (PROMEDIO) | LOCRO DE PAPA (PROMEDIO) | 3.70 | 6.54 | 7.69 | 103.29 | 0.048064 | 0.0455709 |
| MADURO CON QUESO (PROMEDIO) | MADURO CON QUESO (PROMEDIO) | 7.37 | 8.84 | 18.06 | 179.81 | 0 | 0 |
| MAITO (PROMEDIO) | MAITO (PROMEDIO) | 6.72 | 0.68 | 14.50 | 86.62 | 0.2627957 | 0.2170646 |
| MAZAMORRA (PROMEDIO) | MAZAMORRA (PROMEDIO) | 3.53 | 2.94 | 33.01 | 168.47 | 0.178515 | 0.1635239 |
| MENUDO DE CERDO (PROMEDIO) | MENUDO DE CERDO (PROMEDIO) | 11.10 | 7.32 | 6.44 | 135.27 | 0.2772985 | 0.2034139 |
| MOROCHO (PROMEDIO) | MOROCHO (PROMEDIO) | 3.78 | 3.06 | 25.36 | 141.13 | 0.1920202 | 0.1767251 |
| MOTE CASADO (PROMEDIO) | MOTE CASADO (PROMEDIO) | 4.62 | 2.43 | 18.32 | 111.48 | 0.1962106 | 0.126309 |
| MOTE PILLO (PROMEDIO) | MOTE PILLO (PROMEDIO) | 3.92 | 2.72 | 14.97 | 98.44 | 0.1997348 | 0.1637845 |
| MOTE SUCIO (PROMEDIO) | MOTE SUCIO (PROMEDIO) | 3.95 | 18.41 | 16.03 | 244.17 | 0.0831187 | 0.0688238 |
| PAPAS CON CUERO (PROMEDIO) | PAPAS CON CUERO (PROMEDIO) | 3.11 | 5.86 | 5.91 | 86.28 | 0.0596929 | 0.0408371 |
| PAPAS LOCAS (PROMEDIO) | PAPAS LOCAS (PROMEDIO) | 6.83 | 11.53 | 10.80 | 170.10 | 0.1721965 | 0.1209796 |
| PASTEL DE CHOCOLATE (PROMEDIO) | PASTEL DE CHOCOLATE (PROMEDIO) | 8.56 | 8.02 | 63.49 | 364.09 | 0.3991582 | 0.250772 |
| PASTEL DE MAQUEÑO (PROMEDIO) | PASTEL DE MAQUEÑO (PROMEDIO) | 6.51 | 10.27 | 30.87 | 237.23 | 0.0780799 | 0.0564793 |
| PASTEL DE ZANAHORIA (PROMEDIO) | PASTEL DE ZANAHORIA (PROMEDIO) | 7.60 | 8.64 | 63.03 | 361.82 | 0.3869008 | 0.2437626 |
| PESCADO FRITO (PROMEDIO) | PESCADO FRITO (PROMEDIO) | 20.33 | 19.93 | 1.34 | 268.77 | 0.795678 | 0.6852862 |
| PINCHO (PROMEDIO) | PINCHO (PROMEDIO) | 10.29 | 12.54 | 7.10 | 183.91 | 0.3948453 | 0.3347804 |
| PINCHO DE CHONTACURO (PROMEDIO) | PINCHO DE CHONTACURO (PROMEDIO) | 15.75 | 12.83 | 0.00 | 179.25 | 0 | 0 |
| PLÁTANO ASADO CON QUESO (PROMEDIO) | PLÁTANO ASADO CON QUESO (PROMEDIO) | 5.27 | 6.18 | 19.02 | 144.77 | 0.0252131 | 0.0183368 |
| PUCHERO (PROMEDIO) | PUCHERO (PROMEDIO) | 4.22 | 2.79 | 10.49 | 84.32 | 0.1559072 | 0.1330682 |
| QUIMBOLITO (PROMEDIO) | QUIMBOLITO (PROMEDIO) | 7.72 | 20.27 | 47.28 | 395.07 | 0.205244 | 0.1624636 |
| REFRESCO DE AVENA (PROMEDIO) | REFRESCO DE AVENA (PROMEDIO) | 2.62 | 1.07 | 30.00 | 135.88 | 0.1352166 | 0.0870863 |
| SALCHIPAPAS (PROMEDIO) | SALCHIPAPAS (PROMEDIO) | 3.23 | 17.24 | 13.56 | 220.18 | 0.0919489 | 0.0770558 |
| SANCOCHO DE PESCADO (PROMEDIO) | SANCOCHO DE PESCADO (PROMEDIO) | 1.71 | 4.82 | 4.67 | 67.63 | 0.0662123 | 0.0528381 |
| SANGO DE CAMARÓN (PROMEDIO) | SANGO DE CAMARÓN (PROMEDIO) | 3.31 | 0.57 | 22.53 | 109.02 | 0.1057082 | 0.0711228 |
| SECO DE CARNE (PROMEDIO) | SECO DE CARNE (PROMEDIO) | 7.48 | 4.72 | 15.42 | 134.37 | 0.2871855 | 0.2394953 |
| SECO DE CHIVO (PROMEDIO) | SECO DE CHIVO (PROMEDIO) | 4.07 | 3.27 | 21.19 | 132.92 | 0.1708467 | 0.1243117 |
| SECO DE GALLINA CRIOLLA (PROMEDIO) | SECO DE GALLINA CRIOLLA (PROMEDIO) | 7.81 | 5.71 | 13.14 | 137.41 | 0.3078804 | 0.2390416 |
| SECO DE POLLO (PROMEDIO) | SECO DE POLLO (PROMEDIO) | 4.41 | 3.95 | 21.68 | 142.03 | 0.1856283 | 0.1306332 |
| SOPA DE ARROZ CON QUESO (PROMEDIO) | SOPA DE ARROZ CON QUESO (PROMEDIO) | 4.76 | 5.84 | 16.18 | 137.56 | 0.1145864 | 0.0895922 |
| SOPA DE BOLAS DE VERDE (PROMEDIO) | SOPA DE BOLAS DE VERDE (PROMEDIO) | 5.86 | 12.96 | 6.69 | 163.81 | 0.2338873 | 0.1887998 |
| SOPA DE FIDEO CON PAPAS Y QUESO (PROMEDIO) | SOPA DE FIDEO CON PAPAS Y QUESO (PROMEDIO) | 4.17 | 4.96 | 15.04 | 121.76 | 0.0994476 | 0.0740402 |
| SOPA DE MOROCHO (PROMEDIO) | SOPA DE MOROCHO (PROMEDIO) | 4.41 | 5.97 | 12.46 | 120.29 | 0.1806068 | 0.1614116 |
| SOPA DE QUINOA CON HUESO DE CHANCHO (PROMEDIO) | SOPA DE QUINOA CON HUESO DE CHANCHO (PROMEDIO) | 4.16 | 4.36 | 16.34 | 119.35 | 0.1631973 | 0.1019383 |
| SOPA TIMBUSHCA (PROMEDIO) | SOPA TIMBUSHCA (PROMEDIO) | 3.37 | 7.19 | 6.87 | 104.17 | 0.1134743 | 0.0938591 |
| TALLARÍN DE POLLO (PROMEDIO) | TALLARÍN DE POLLO (PROMEDIO) | 5.54 | 5.07 | 19.60 | 147.43 | 0.2345928 | 0.1681895 |
| TAMAL DE CERDO (PROMEDIO) | TAMAL DE CERDO (PROMEDIO) | 4.91 | 8.35 | 14.04 | 148.90 | 0.221946 | 0.1775278 |
| TAMAL DE POLLO (PROMEDIO) | TAMAL DE POLLO (PROMEDIO) | 4.54 | 8.02 | 14.44 | 145.79 | 0.2064561 | 0.1629167 |
| TAPAO ARRECHO (PROMEDIO) | TAPAO ARRECHO (PROMEDIO) | 10.27 | 12.52 | 5.52 | 176.40 | 0.4498568 | 0.3250196 |
| TAPAO DE PESCADO (PROMEDIO) | TAPAO DE PESCADO (PROMEDIO) | 5.10 | 1.65 | 9.23 | 68.33 | 0.1944668 | 0.1616505 |
| TORTA DE FRUTA DEL PAN (PROMEDIO) | TORTA DE FRUTA DEL PAN (PROMEDIO) | 7.00 | 7.84 | 14.98 | 156.12 | 0.1388796 | 0.1155787 |
| TORTILLA DE PAPA (PROMEDIO) | TORTILLA DE PAPA (PROMEDIO) | 6.44 | 10.09 | 14.62 | 173.44 | 0.0016728 | 0.0016728 |
| TORTILLAS DE MAÍZ DE TIESTO (PROMEDIO) | TORTILLAS DE MAÍZ DE TIESTO (PROMEDIO) | 8.34 | 12.78 | 38.12 | 295.86 | 0.1789473 | 0.1507517 |
| TOSTADO Y HABAS CALPU (PROMEDIO) | TOSTADO Y HABAS CALPU (PROMEDIO) | 3.12 | 0.67 | 21.07 | 100.26 | 0.1168634 | 0.0925262 |
| TRIPA MISHQUI (PROMEDIO) | TRIPA MISHQUI (PROMEDIO) | 5.17 | 1.85 | 15.83 | 99.04 | 0.0763486 | 0.0621249 |
| VICHE DE CAMARÓN (PROMEDIO) | VICHE DE CAMARÓN (PROMEDIO) | 3.75 | 3.36 | 10.11 | 81.63 | 0.0755964 | 0.0604387 |
| VICHE DE MONDONGO (PROMEDIO) | VICHE DE MONDONGO (PROMEDIO) | 4.07 | 3.29 | 8.14 | 76.15 | 0.0620544 | 0.050159 |
| VICHE DE PESCADO (PROMEDIO) | VICHE DE PESCADO (PROMEDIO) | 2.67 | 3.42 | 5.71 | 61.81 | 0.1114285 | 0.0889968 |
| YAHUARLOCRO (PROMEDIO) | YAHUARLOCRO (PROMEDIO) | 6.51 | 3.77 | 6.04 | 83.22 | 0.0660723 | 0.052336 |
|  | |  |  |  |  |  |  |
| BURGER KING HAMBURGUESA DE QUESO | BURGER KING, CHSBRGR | 14.57 | 14.81 | 23.71 | 286 | 0.604 | 0.467 |
| BURGER KING HAMBURGUESA PROMEDIO | BURGER KING,HAMBURGER | 14.85 | 10.55 | 26.76 | 261 | 0.553 | 0.385 |
| BURGER KING NUGGETS DE POLLO | BURGER KING,CHICK STRIPS | 15.42 | 20.89 | 17.55 | 320 | 0.604 | 0.467 |
| BURGER KING PAPAS FRITAS | BURGER KING,FRENCH FR | 3.23 | 12.48 | 38.7 | 280 | 0.464 | 0.262 |
| BURGER KING SANDUCHE DE PESCADO ORIGINAL | BURGER KING,ORIGINAL CHICK SNDWCH | 12.14 | 14.69 | 26.22 | 286 |  |  |
| BURGER KING SANDUCHE DE PESCADO PREMIUM | BURGER KING,PREMIUM FISH SNDWCH | 10.29 | 12.45 | 26.69 | 260 | 0.417 | 0.282 |
| BURGER KING WHOPPER SIN QUESO | BURGER KING,WHOPPER,NO CHS | 10.74 | 12.84 | 18.55 | 233 | 0.177 | 0.118 |
| BURGER KING WHOPPER CON QUESO | BURGER KING,WHOPPER,W/ CHS | 11.19 | 15.33 | 16.7 | 250 | 0.826 | 0.661 |
| BURGER KING WHOPPER DOBLE CON QUESO | BURGER KING,DOUBLE WHOPPER,W/ CHS | 14.47 | 17.06 | 13.52 | 266 | 0.852 | 0.682 |
| BURGER KING WHOPPER DOBLE SIN QUESO | BURGER KING,DOUBLE WHOPPER,NO CHS | 13.94 | 15.66 | 13.74 | 252 | 0.843 | 0.675 |
| HAMBURGUESA | FT FDS, HAMBRGER; SIN, LRG PATTY; W/ CONDMNTS, VEG, MAYO | 11.34 | 12.37 | 17.33 | 226 | 0.705 | 0.564 |
| HAMBURGUESA DOBLE | FAST FOODS,HAMBURGER; DOUBLE,LRG PATY; W/ CONDMNT,VEG & MAYO | 13.94 | 15.66 | 13.74 | 252 | 0.796 | 0.637 |
| HOT DOG PROMEDIO | FAST FOODS,HOTDOG,PLAIN | 10.6 | 14.84 | 18.4 | 247 | 0.812 | 0.65 |
| KFC ALA CRISPY | KENTUCKY FRIED CHICK CRISPY,WING,MEAT & SKN W/ BREADING | 20.8 | 23 | 11.66 | 337 | 0.789 | 0.631 |
| KFC ALA ORIGINAL | KENTUCKY FRIED CHICK ORG RECIPE,WING,MEAT & SKN W/ BRDING | 21.94 | 18.14 | 8.69 | 286 | 0.657 | 0.526 |
| KFC BBQ HOT WINGS | KENTUCKY FRIED CHICK CRISPY,WING,MEAT & SKN W/ BREADING | 20.8 | 23 | 11.66 | 337 | 0.743 | 0.473 |
| KFC ENSALADA CESAR CON POLLO | MCDONALD'S,CAESAR SALAD W/ CRISPY CHICK | 8.32 | 5.31 | 6.2 | 113 | 0.796 | 0.637 |
| KFC ENSALADA DE COL | KENTUCKY FRIED CHICK,COLESLAW | 0.91 | 8.64 | 15.65 | 144 | 0.796 | 0.637 |
| KFC HAMBURGUESA DE POLLO | BURGER KING,ORIGINAL CHICK SNDWCH | 12.14 | 14.69 | 26.22 | 286 | 0.56 | 0.328 |
| KFC HOT WINGS | KENTUCKY FRIED CHICK CRISPY,WING,MEAT & SKN W/ BREADING | 20.8 | 23 | 11.66 | 337 | - | - |
| KFC MUSLO CRISPY | KENTUCKY FRIED CHICK CRISPY,THIGH,MEAT & SKN W/ BREADING | 17.17 | 22.14 | 10.3 | 309 | 0.025 | 0.017 |
| KFC MUSLO ORIGINAL | KENTUCKY FRIED CHICK,ORG RECIPE,THIGH,MEAT & SKN W/ BRDING | 18.75 | 17.38 | 7.16 | 260 | - | - |
| KFC PAPAS FRITAS | KENTUCKY FRIED CHICK,POTATO WEDGES,ANALYZED PRIOR 2007 | 4.05 | 14.77 | 33.55 | 283 | 0.53 | 0.33 |
| KFC PECHUGA CRISPY | KENTUCKY FRIED CHICK CRISPY,BREAST,MEAT & SKN W/ BRDING | 21.24 | 16.55 | 8.47 | 268 | - | - |
| KFC PECHUGA ORIGINAL | KENTUCKY FRIED CHICK ORIG RECIPE,BREAST,MEAT & SKN W/ BRDING | 22.54 | 12.03 | 5.53 | 221 | 0.39 | 0.245 |
| KFC PIERNA CRISPY | KENTUCKY FRIED CHICK CRISPY,DRUMSTK,MEAT & SKN W/ BRDING | 20.62 | 17.73 | 7.96 | 274 | 0.36 | 0.237 |
| KFC PIERNA ORIGINAL | KENTUCKY FRIED CHICK ORG RECIPE,DRUMSTK,MEAT & SKN W/ BRDING | 22.3 | 14.22 | 5.39 | 239 | 0.42 | 0.283 |
| KFC POP CORN INDIVIDUAL | KENTUCKY FRIED CHICK,POPCORN CHICK | 17.67 | 21.74 | 21.18 | 351 | 0.263 | 0.185 |
| MCDONALD'S BIG MAC | MCDONALD'S,BIG MAC | 11.82 | 14.96 | 20.08 | 257 | - | - |
| MCDONALD'S CUARTO DE LIBRA | MCDONALD'S,QUARTER POUNDER | 14.1 | 11.55 | 22.17 | 244 | - | - |
| MCDONALD'S CUARTO DE LIBRA CON QUESO | MCDONALD'S,QUARTER POUNDER W/ CHS | 14.59 | 14.22 | 19.95 | 258 | - | - |
| MCDONALD'S HAMBURGUESA CON QUESO | MCDONALD'S, CHEESEBURGER | 12.97 | 11.79 | 27.81 | 263 | - | - |
| MCDONALD'S HAMBURGUESA PROMEDIO | MCDONALD'S,HAMBURGER | 12.92 | 10.09 | 30.28 | 264 | - | - |
| MCDONALDS MCFLURRY CON GALLETAS OREO | MCDONALD'S,MCFLURRY W/ OREO COOKIES | 3.97 | 5.66 | 25.55 | 165 | - | - |
| MCDONALD'S PAPAS FRITAS | MCDONALD'S,FRENCH FR | 3.41 | 15.47 | 42.58 | 323 | 0.953 | 0.697 |
| NUGGETS DE POLLO | CHICKEN NUGGETS,FRZ,CKD | 15.27 | 19.82 | 14.09 | 296 | 0.752 | 0.491 |
| PIZZA PEPPERONI, SALCHICHA, CHAMPIÑONES, CARNE RES, CARNE CERDO, PIMIENTO, PROMEDIO | FAST FD,PIZZA CHAIN,14" PIZZA,MEAT & VEG TOPPING,REG CRUST | 11.02 | 10.9 | 25.38 | 244 | 0.901 | 0.729 |
| PIZZA DE QUESO | FAST FD,PIZZA CHAIN,14" PIZZA,CHS TOPPING,REG CRUST | 11.39 | 9.69 | 33.33 | 266 | 0.17 | 0.265 |
| PIZZA PEPPERONI | FAST FD,PIZZA CHAIN,14" PIZZA,PEPPERONI TOPPING,REG CRUST | 11.74 | 11.91 | 31.98 | 282 | - | - |
| PIZZA PEPPERONI Y CHAMPIÑONES PROMEDIO | FAST FD,PIZZA CHAIN,14" PIZZA,PEPPERONI TOPPING,REG CRUST | 11.74 | 11.91 | 31.98 | 282 | 1.09 | 0.94 |
| SUBWAY ATÚN EN PAN BLANCO CON LECHUGA Y TOMATE | SUBWAY,TUNA SUB ON WHITE BREAD,W/ LETTUCE & TOMATO | 12.33 | 12.04 | 15.95 | 221 | 0.762 | 0.593 |
| SUBWAY JAMÓN PAN BLANCO CON TOMATE Y LECHUGA | SUBWAY,COLD CUT SUB ON WHITE BREAD,W/ LETTUCE & TOMATO | 10.52 | 10.04 | 20.43 | 214 | 0.96 | 0.84 |
| SUBWAY ROAST BEEF PAN BLANCO CON TOMATE Y LECHUGA | SUBWAY,RST BF SUB ON WHITE BREAD,W/ LETTUCE & TOMATO | 12.17 | 2.73 | 20.34 | 155 | 0.98 | 0.95 |
| TACO BELL BURRITO DE CARNE | TACO BELL,BURRITO SUPREME W/ BF | 8.05 | 8.05 | 21.07 | 189 | 1.06 | 1.01 |
| TACO BELL BURRITO DE FRIJOLES | TACO BELL,BEAN BURRITO | 7.35 | 6.05 | 31.23 | 209 |  |  |
| TACO BELL NACHOS | TACO BELL,NACHOS | 5.15 | 22.17 | 36.38 | 366 | 0.803 | 0.661 |
| TACO BELL TACO ORIGINAL | TACO BELL,ORIGINAL TACO W/ BF,CHS & LETTUCE | 8.86 | 12.7 | 19.85 | 229 | 0.936 | 0.811 |
| TACO BELL BURRITO DE POLLO | TACO BELL,BURRITO SUPREME W/ CHICK | 9.84 | 6.42 | 20.51 | 179 | 0.664 | 0.522 |

**Supplementary Table 1.** This table contains nutritional information and quantification of phenylalanine and tyrosine of the most common available products in the Latin American region.
